# Biotransformation of triterpenoid ganoderic acids from exogenous diterpene dihydrotanshinone I in the cultures of *Ganoderma sessile*

**DOI:** 10.1186/s12934-023-02156-5

**Published:** 2023-07-28

**Authors:** Xinwei Wang, Haibo Wu, Ka Hong Wong, Yixuan Wang, Baixiong Chen, Kun Feng

**Affiliations:** 1 School of Bioengineering, Zunyi Medical University, Jinwan Road No. 368, Zhuhai, 519090 Guangdong China; 2 State Key Laboratory of Quality Research in Chinese Medicine, Institute of Chinese Medical Sciences, University of Macau, Macau, 999078 China

**Keywords:** *Ganoderma sessile*, Dihydrotanshinone I, Biotransformation, Ganoderic acids

## Abstract

**Background:**

Triterpenoids have shown a wide range of biological activities including antitumor and antiviral effects. Typically, triterpenes are synthesized through the mevalonate pathway and are extracted from natural plants and fungi. In this work, triterpenoids, ganoderic acids (GAs) were discovered to be produced via biotransformation of a diterpene, 15,16-dihydrotanshinone I (DHT) in the liquid cultured *Ganoderma sessile* mycelium.

**Results:**

Firstly, the biotransformation products, two rare GAs were isolated and purified by column chromatography, and characterized using HR-ESI-MS spectrometry and NMR spectrometry. The two compounds were Lanosta-7,9(11),24-trien-15*α*,22,*β*-diacetoxy-3*β*-hydroxy-26-oic acid (LTHA) and Lanosta-7,9(11),24-trien-15*α*,22,*β*-diacetoxy-3*β*-carbonyl-26-oic acid (LTCA). Then, transcriptome and proteome technologies were employed to measure the expression of mRNA and protein, which further confirmed that triterpenoid GAs could be transformed from exogenous diterpenoid DHT. At the molecular level, we proposed a hypothesis of the mechanism by which DHT converted to GAs in *G. sessile* mycelium, and the possible genes involved in biotransformation were verified by RT-qPCR.

**Conclusions:**

Two rare GAs were obtained and characterized. A biosynthetic pathway of GAs from DHT was proposed. Although the synthetic route was not confirmed, this study provided important insights into omics resources and candidate genes for studying the biotransformation of diterpenes into triterpenes.

**Supplementary Information:**

The online version contains supplementary material available at 10.1186/s12934-023-02156-5.

## Background

**Fig. 1:**
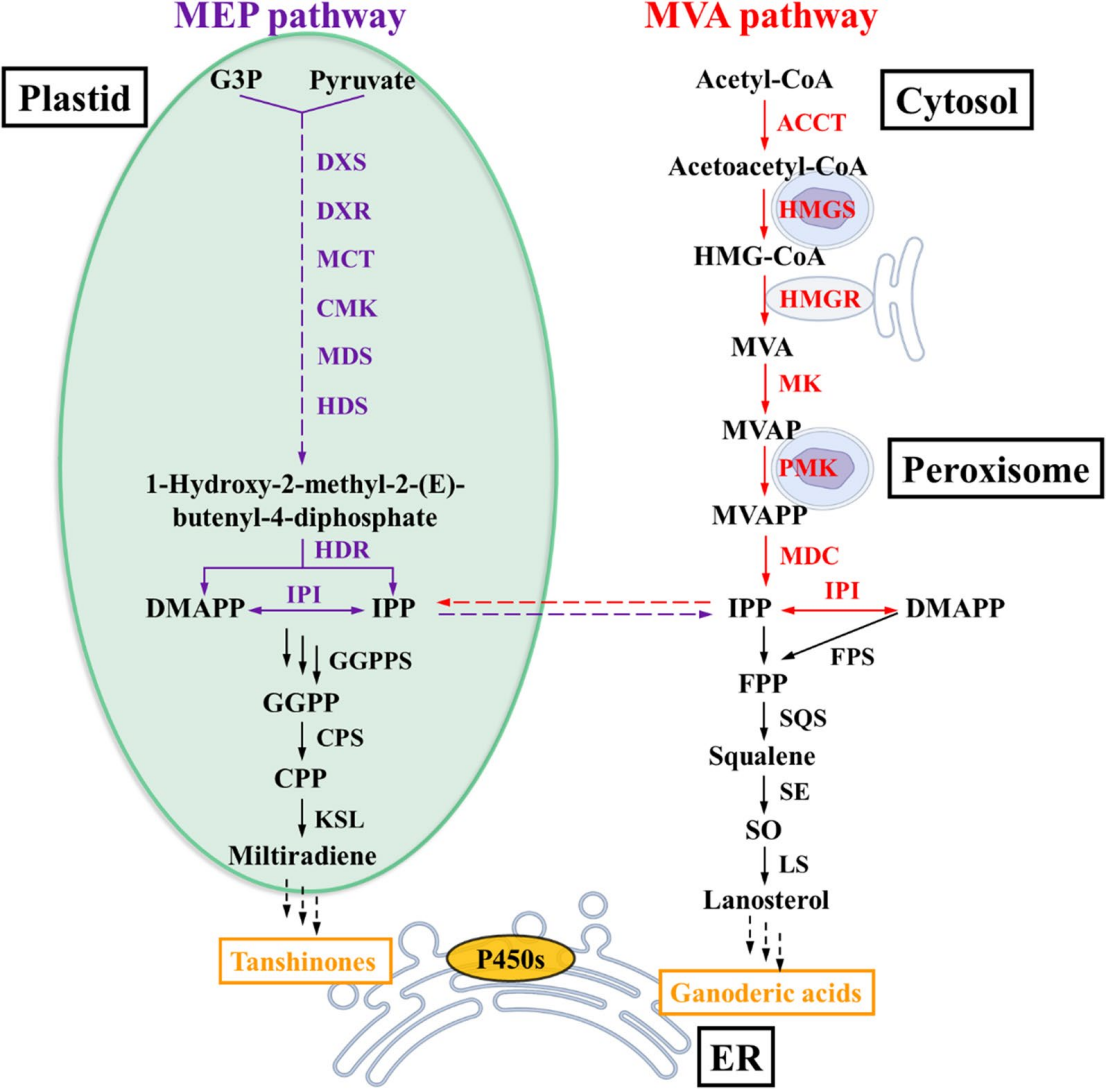
Subcellular compartmentalization of the MVA and MEP pathway. G3P, glyceraldehyde-3-phosphate; DXS, 1-deoxy-d-xylulose 5-phosphate synthase; DXR, 1-deoxy- d-xylulose 5-phosphate reductoisomerase; MCT, 4-diphosphocytidyl-2-C-methyl d-erythritol synthase; CMK, CDP-ME2P kinase; MDS, 2-C-methyl- d-erythritol 2, 4-cyclodiphosphate synthase; HDS, HMBPP synthase; HDR, HMBPP reductase; IPI, isopentenyl pyrophosphate isomerase; GGPPS, GGPP synthase; CPP, copalyl diphosphate; CPS, CPP synthases; KSL, kaurene synthase-like diterpene synthases; ACCT, acetoacetyl-CoA thiolase; HMG-CoA, 3-hydroxy-3-methylglutaryl-CoA; HMGS, HMG-CoA synthase; HMGR, HMG-CoA reductase; MK, mevalonate kinase; MVAP, mevalonate phosphate; PMK, phosphomevalonate kinase; MVAPP, mevalonate pyrophosphate; MDC, pyrophosphate decarboxylase; FPP, farnesyl pyrophosphate; SE, squalene epoxidase

Triterpenoids represent a major group of compounds that can be extracted from nature plants and fungi, have aroused attention for showing broad-spectrum activities including antitumor, antibacterial, anti-HIV-1, hepatoprotection, etc. [1, 2]. In general, triterpenoids are biosynthesized from the universal triterpenoid compound precursors isopentenyl diphosphate (IPP) and dimethylallyl diphosphate (DMAPP), which are largely produced via mevalonate/isoprenoid (MVA) pathway in the cytoplasm. Then, IPP and DMAPP are catalyzed by farnesyl pyrophosphate synthase (FPS) to generate FPP, which is further catalyzed into squalene in the present of squalene synthase (SQS) [3–6]. Finally, the precursor of steroids and saponins, lanosterol are produced under the effect of squalene 2,3-epoxidase (SO) and lanosterol synthase (LS). Similarly, the synthesis of diterpenes start from the IPP and DMAPP via 2-C-methyl-d-erythritol 4-phosphate (MEP) pathways in the plastid. Following, DMAPP and IPP are condensed to form the diterpenoid precursor (*E*,*E*,*E*)-geranylgeranyl diphosphate (GGPP) by GGPPS catalyzation. After that, a variety of diterpenes is obtained through the further catalyzation by different enzymes [7–9]. Although diterpenes and triterpenes are biosynthesized via different pathways, both synthetic pathways passed through the step of forming universal precursors IPP and DMAPP (Fig. 1). It is of great interest to investigate whether triterpenes can be obtained from the biosynthesis of diterpenes directly.

In traditional Chinese medicine, *Ganoderma* is the source of ganoderic acids (GAs), a class of highly oxygenated lanostane-type triterpenoids [10], multi-functionality has been reported for GAs [2]. Nowadays, major method to obtain GAs is via the artificial cultivation of the *Ganoderma* mushroom primordium and fruiting bodies. However, the *Ganoderma* takes a long time to grow and the content of the GAs is low. Furthermore, the extraction procedures of GAs are complicated, which highly hamper the clinical applications of GAs [11–13]. Biotransformation is an important technical means for discovery of new active ingredients. A microbial metabolic process can modify the product-specific structure by producing enzymes, posing the advantages of mild conditions, simplicity and high efficiency [14, 15]. If biotransformation technique can be applied for GAs production, it can largely reduce the time and cost.

Tanshinones, the main ingredient in Chinese herbal medicine *Salvia miltiorrhiza* Bunge, has been shown to undergo various transformations [16, 17]. For examples, tanshinone IIA was converted into tanshisorbicin via a [4+2] cycloaddition reaction by the fungus *Hypocrea* sp. [18]. In another study, the use of *Cunninghamella elegans* led to the identification of two novel glycosylated derivatives resulting from the biotransformation of tanshinone IIA [19]. These findings suggested that tanshinones possess significant reactivity for biotransformation processes, making them potential candidates of interest. Consequently, tanshinones were chosen as the substrates for biotransformation screening experiments and 15,16-dihydrotanshinone I (DHT), an abietane diterpenes [20], was selected for further biotransformation study.

In this work, a rapid and inexpensive method to produce GAs was discovered by adding the substrate DHT into the liquid cultured *Ganoderma sessile* mycelium for biotransformation. Studying the molecular mechanism behind the biotransformation of DHT into GAs from the molecular level was conducted using transcriptome and proteome technologies. Up to now, there is only few reports focusing on the structural transformation between diterpene and triterpenoid compounds. Through the comprehensive analysis of the transcriptome and proteome of mycelium before and after biotransformation, our data presented here provide insight into omics resources and genes involved in diterpene to triterpene biotransformation in *G. sessile* mycelium.

## Results

### Identification of biotransformation

**Fig. 2:**
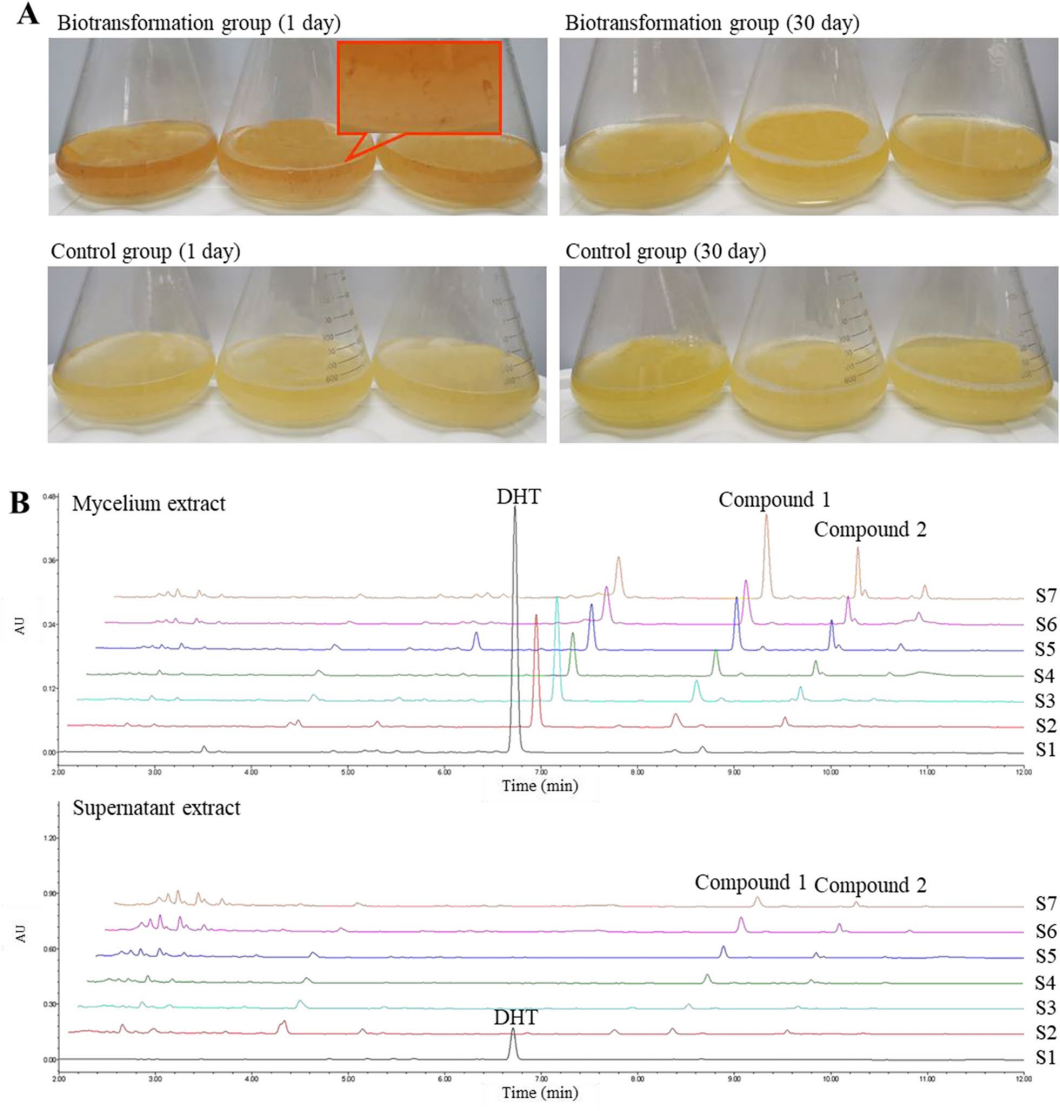
**A** Growth state of *G. sessile* mycelium medium; **B** UPLC chromatogram of biotransformation process in experimental group. S1–S7: 1, 5, 10, 15, 20, 25 and 30 days of biotransformation, respectively

**Fig. 3:**
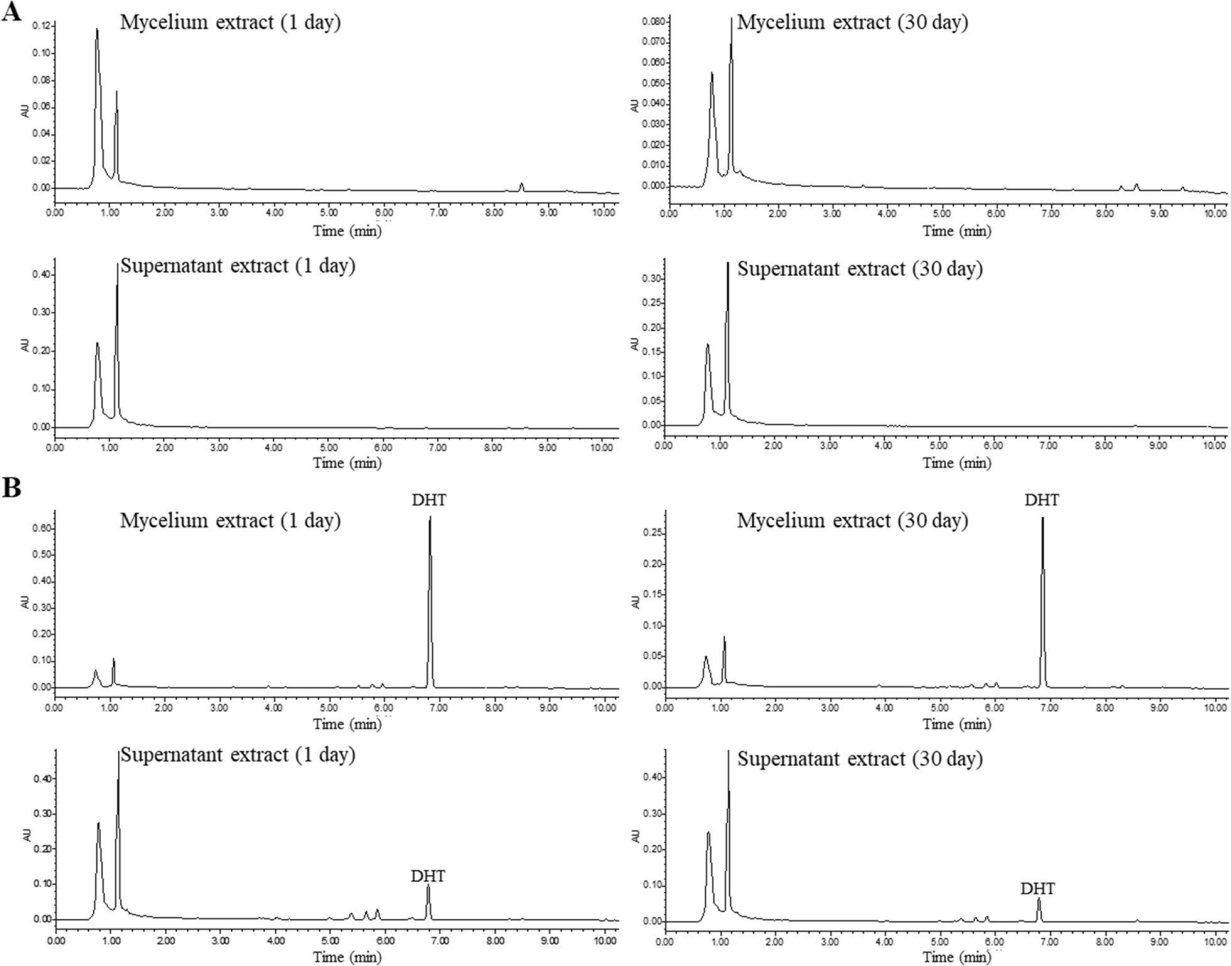
**A** UPLC chromatogram of *G. sessile* culture; **B** UPLC chromatogram of inactivated *G. sessile* culture

The transformation of DHT was observed through changes in color, as it was red and adsorbed on the mycelium. After a 30-day transformation period, the color of the mycelium returned to normal (Fig. 2A). The biotransformation process of DHT in *G. sessile* mycelium medium was monitored and confirmed by using ultra performance liquid chromatography (UPLC). Chromatograms of culture medium and mycelium extract of E group were shown in Fig. 2B. During biotransformation, the peak of substrate DHT (6.8 min) decreased gradually, and two new peaks (8.2 min and 9.4 min) were detected in the extract of *G. sessile* mycelium. These two peaks were not found in the C group mycelium and culture medium (Additional file 1: Figure S1). To confirm whether compound 1 and 2 were formed through the biotransformation of DHT, G. sessile mycelium was subjected to high-temperature inactivation, followed by the addition of DHT to the medium for a 30-day culture period. UPLC analysis detected the continued presence of the DHT substrate in the sample, while the two biotransformation products were not detected in either the mycelia or the supernatant (Fig. 3), indicating the biotransformation is highly related to the activity of the mycelium.

After biotransformation, the intracellular enzymes of *G. sessile* can transform DHT to produce GA LTHA and GA LTCA. However, the DHT was not altered after 72 h of incubation in enzyme extracts of *G. sessile* pure cultures (Additional file 1: Figure S2). Addition of DHT enabled mycelium to obtain the activity of biotransformation to produce GA LTHA and GA LTCA, which was completed by intracellular enzymes.

### Isolation and identification of biotransformation products

**Fig. 4:**
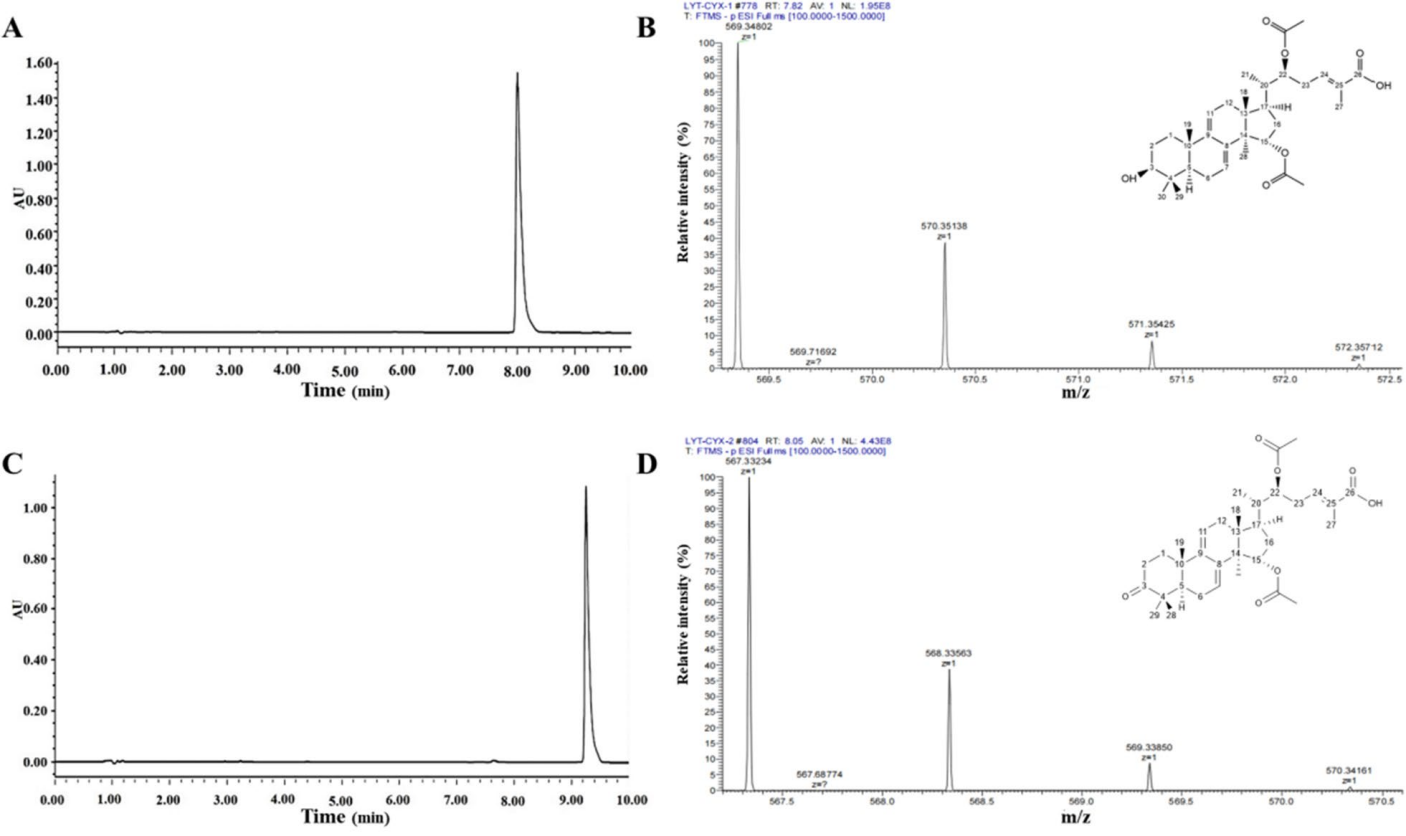
**A** UPLC chromatogram of compound 1; **B** High-resolution mass spectrum of compound 1; **C** UPLC chromatogram of compound 2; **D** High-resolution mass spectrum of compound 2

These two compounds were separated by column chromatography and characterized by using HR-ESI-MS spectrometer and NMR spectrometer (Fig. 4 and Additional file 1: Figure S3). The identification data are as follows:

Compound 1 (8.4 min): white solids; HR-ESI-MS m/z calcd. for [M+H]^+^: 569.34802 (Anal. Calcd. for C_34_H_50_O_7_: 569.34838). Additional file 1: Table S1 shows the ^1^H-NMR and ^13^C-NMR data (CDCl_3_, 600 MHz). Compound 1 was identified as lanosta-7,9(11),24-trien-15*α*,22,*β*-diacetoxy-3*β*-hydroxy-26-oic acid (GA LTHA) [21].

Compound 2 (9.5 min): white solids; HR-ESI-MS m/z calcd. for [M+H]^+^: 567.33234 (Anal. Calcd. for C_34_H_48_O_7_: 567.33273). Additional file 1: Table S1 shows ^1^H-NMR and ^13^C-NMR data (CDCl_3_, 600 MHz). Compound 2 was identified as lanosta-7,9(11),24-trien-15*α*,22,*β*-diacetoxy-3*β*-carbonyl-26-oic acid (GA LTCA) [22].

### Transcriptome analysis and functional classification

**Fig. 5:**
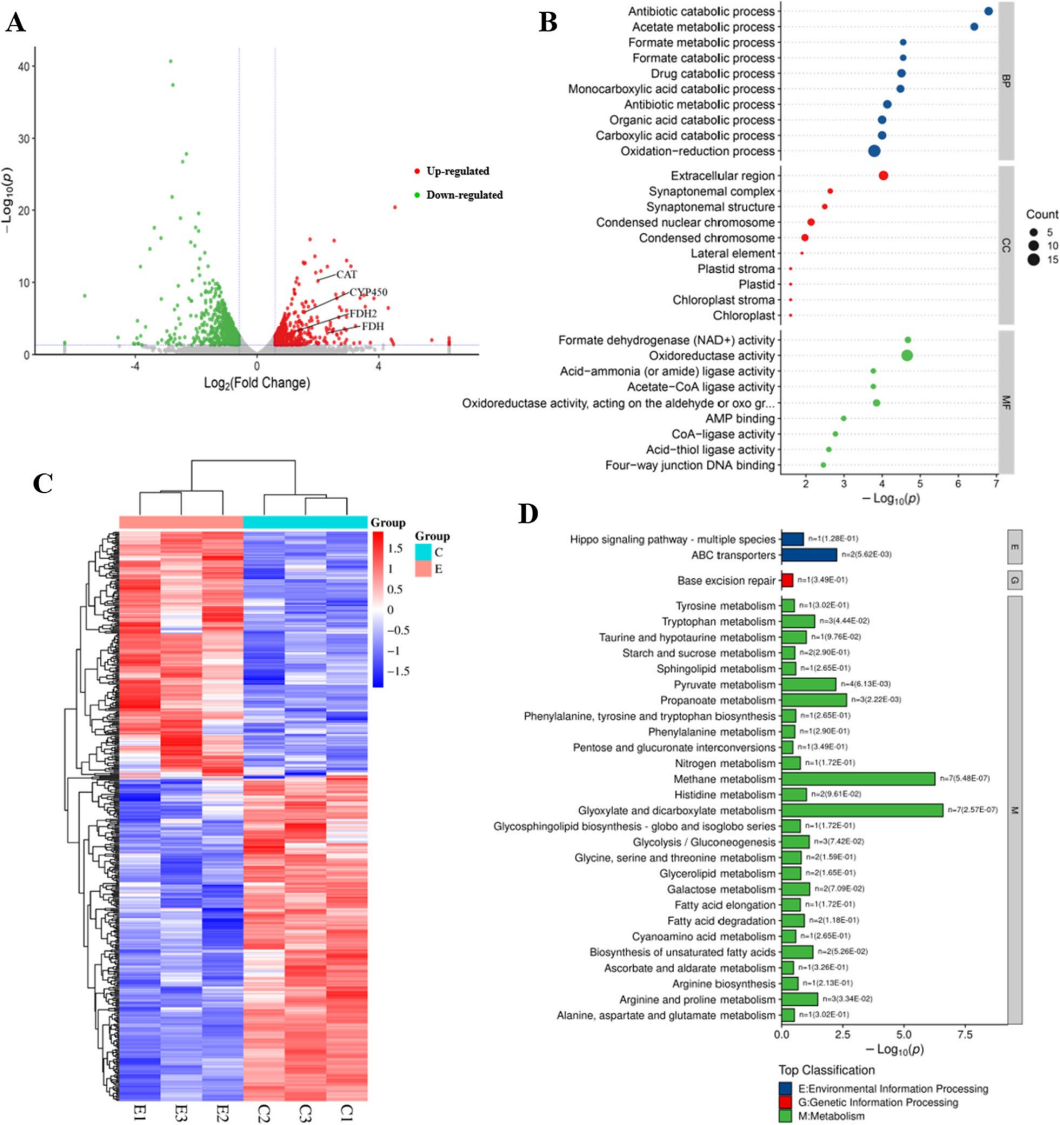
**A** Volcano plot of DEGs; **B** GO analysis of the DEGs (Top 30); **C** Heat map of DEGs; **D** KEGG analysis of the DEGs (Top 30)

The resulting *G. sessile* transcriptome contained 67,230 transcripts and 14,298 unigenes (Additional file 1: Table S2). Differentially expressed genes (DEGs) in the transcriptome were compared and identified. Under the threshold value of |log_2_ FC (fold change) |> 1 and *p* < 0.05. Among these unigenes, 492 were identified as DEGs, of which 213 upregulated and 279 downregulated (Fig. 5A), and Fig. 5C shows the heat map of cluster analysis for DEGs.

After GO enrichment analysis, DEGs were arranged into three categories: biological process (BP), cellular component (CC), and molecular function (MF). In the BP category, the first three subcategories appeared: antibiotic catabolic process, acetate metabolic process and formate metabolic process; while in the CC category, extracellular region, synaptonemal complex, synaptonemal structure were the top three subcategories. For the MF category, the formate dehydrogenase (NAD^+^) active, oxidoreductase activity, and acid-ammonia (or amide) ligase activity were the top three subcategories (Fig. 5B). In KEGG pathway analysis of DEGs, 27 of the top 30 most significantly enriched pathways belonged to metabolism, among which methane metabolism, glyoxylate and dicarboxylate metabolism, propanoate metabolism signaling pathways were the most significantly enriched (Fig. 5D).

### Quantitative analysis and functional classification of proteome

**Fig. 6:**
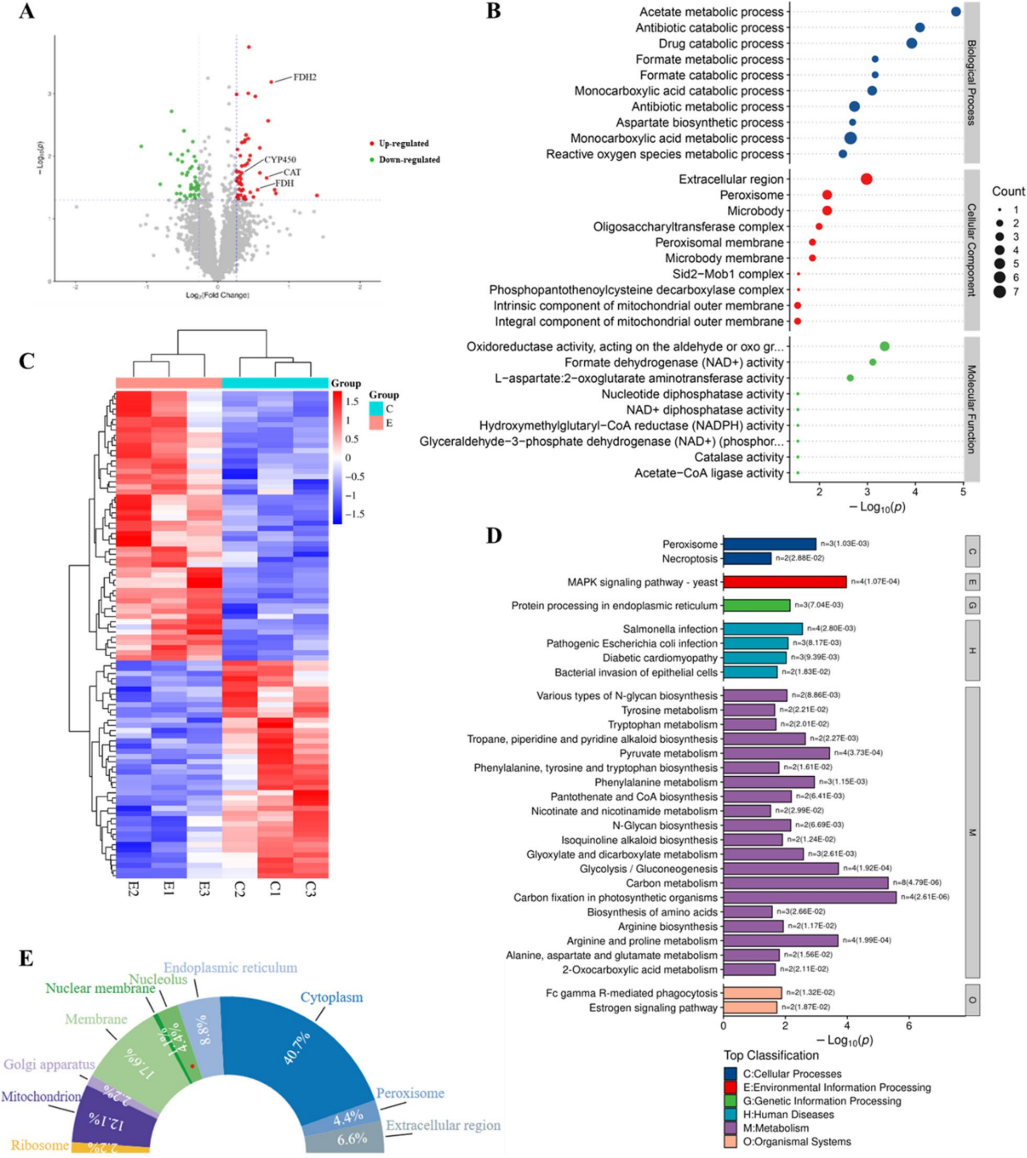
**A** Volcano plot of DEPs; **B** GO analysis of the DEPs (Top 30); **C** Heat map of DEPs; **D** KEGG analysis of the DEPs (Top 30); **E** Subcellular localization analysis of cellular components of DEPs

In total, 16,457 unique peptides, 2606 proteins and 2588 quantified proteins were determined via proteomic analysis. Comparative analysis to identify the differentially expressed proteins (DEPs) between E group and C group under the threshold value of |FC|> 1.2 and *p* < 0.05. Among these 2606 proteins, 94 were identified as DEPs, including 52 upregulated and 42 downregulated (Fig. 6A), and Fig. 6C shows the heat map of cluster analysis for DEPs.

From GO analysis, BP category showed that most DEPs, which were involved in acetate metabolic process, antibiotic catabolic process and drug catabolic process. The highest proportions of DEPs in the CC category were involved in extracellular region, peroxisome and microbody, and the oxidoreductase activity while formate dehydrogenase (NAD^+^) activity, showed the highest portions of DEPs in the MF category (Fig. 6B). Similar to the transcriptome, KEGG pathway analysis revealed that many DEPs were enriched in metabolism, and the three most enriched pathways were carbon fixation in photosynthetic organisms, carbon metabolism and MAPK signaling pathways (Fig. 6D). Moreover, the subcellular localization analysis and annotation of DEPs were performed by analyzing the CC of the GO database (Fig. 6E). The DEPs were mainly distributed in cytoplasm (40.66%), membrane (17.58%), mitochondrion (12.09%) and endoplasmic reticulum (8.79%), which is theoretically the main location of GAs biosynthesis.

### Analysis of correlations between transcriptome and proteome data, RT-qPCR validation

**Fig. 7:**
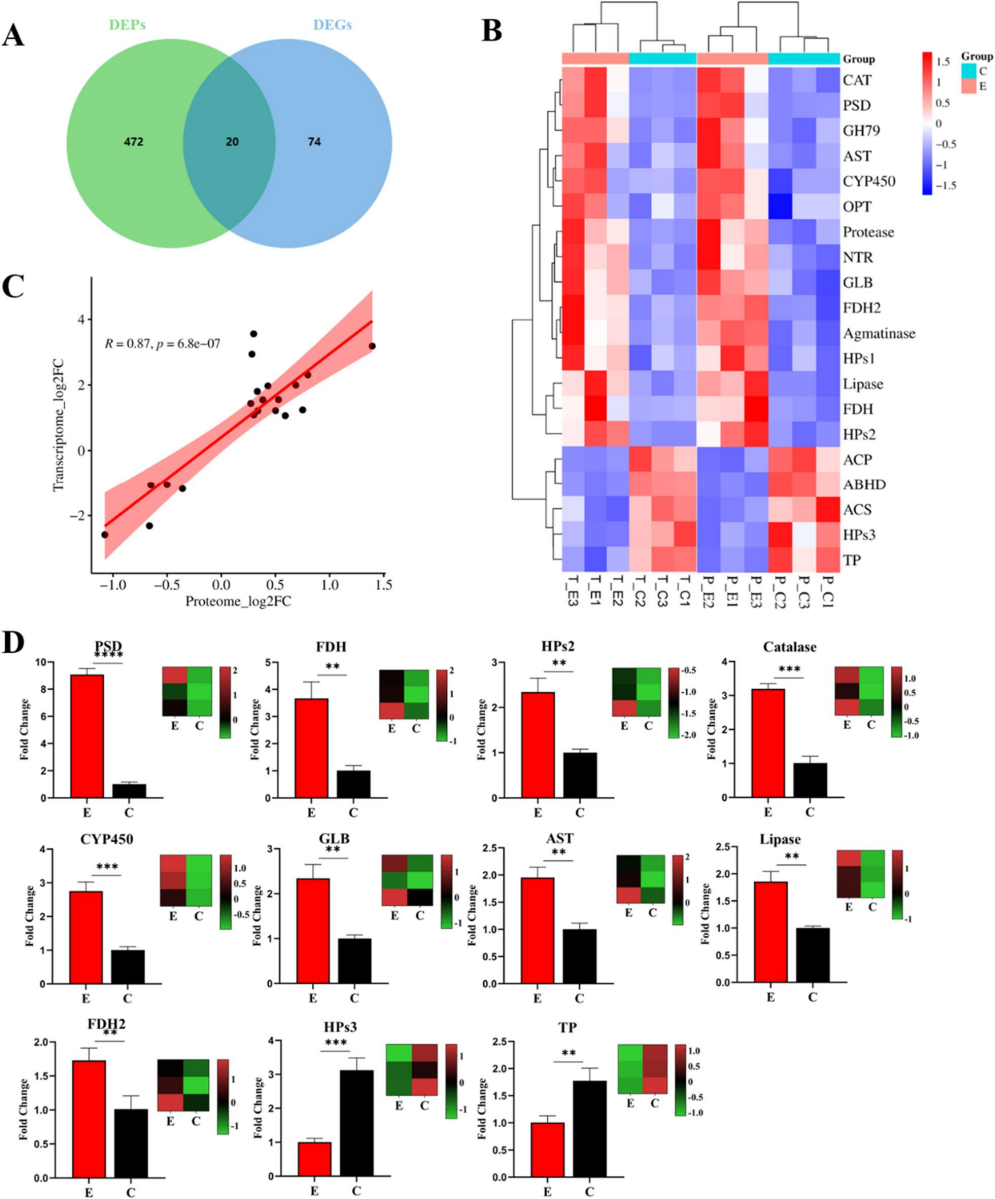
**A** Venn diagram of DEGs/DEPs comparisons between transcriptome and proteome; **B** Heat map of DEGs/DEPs; **C** Correlation analysis of DEGs and DEPs; **D** RT-qPCR validation of transcriptome data. **p < 0.01, ***p < 0.001, ****p < 0.0001; Transcriptome fold changes were calculated using z-score normalization

A Venn diagram was produced for the biotransformation and control group candidate DEGs and DEPs, and there were 20 differentially expressed genes (Fig. 7A). Cluster analysis of the DEGs/DEPs shared by the E group and the C group in the transcriptome and proteome determined that there were 15 upregulated and 5 downregulated genes between the mRNA and protein, the DEGs were positively correlated with the expression levels of their translated DEPs, as show in Table 1. Based on Pearson’s correlation coefficient analysis, a relatively higher correlation (R = 0.87) was detected between the transcriptome and proteome (Fig. 7C).

**Table 1:** DEGs shared by transcriptome and proteome

Gene ID	Abbreviation	Description	mRNA^a^	Protein^b^
DN548	HPs1	Hypothetical protein GSI_03660	11.7786	1.2301
DN1857	PSD	Phosphatidylserine decarboxylase-domain-containing protein	9.0980	2.6276
DN4127	Protease	Protease	7.6715	1.2167
DN9297	FDH	NAD-dependent formate dehydrogenase	4.9531	1.7392
DN3060	CAT	Catalase	3.9901	1.6108
DN3658	Lipase	Lipase	3.9232	1.3476
DN5234	GH79	Glycoside hydrolase family 79 protein	3.4940	1.2591
DN1355	Agmatinase	Agmatinase	2.9359	1.4422
DN3779	CYP450	Cytochrome P450	2.9209	1.3040
DN1775	OPT	Oligopeptide transporter like protein	2.7056	1.2076
DN640	FDH2	NAD-dependent formate dehydrogenase	2.3633	1.6835
DN2099	AST	Aspartate aminotransferase	2.3265	1.4151
DN733	HPs2	Hypothetical protein GSI_02054 (NAD(P) binding site)	2.3225	1.2628
DN13806	NTR	Nitroreductase	2.1282	1.2310
DN3099	GLB	Globin-like protein	2.0911	1.5061
DN646	ACS	Acetyl-coenzyme a synthetase	0.4822	0.7068
DN3490	ABHD	Alpha/Beta hydrolase protein	0.4778	0.6374
DN1019	HPs3	Hypothetical protein GSI_08980 (ATP binding site)	0.4424	0.7799
DN891	TP	Transport protein (ATP binding site)	0.2011	0.6314
DN850	ACP	Acid proteinase	0.1664	0.4746

^a^Transcriptome fold change

^b^Proteome fold change

Furthermore, Real-time quantitative PCR (RT-qPCR) detection showed that the expression levels of 11 DEGs in E and C groups were significantly different (*p* < 0.05), of which 9 were upregulated DEGs while 2 were downregulated DEGs (Fig. 7D). The expression trend of DEGs in RT-qPCR was consistent with the results of transcriptome analysis, and the gene-specific primers are shown in Additional file 1: Table S4.

## Discussion

Currently, GA LTHA and GA LTCA were mainly isolated from *Ganoderma lucidum* and *Ganoderma orbiforme*, respectively [21, 22]. However, there are no reports in the literature regarding the isolation of GA LTHA and GA LTCA from *G. sessile*. In order to validate the credibility of our findings, we conducted thorough investigations. Our analysis confirmed that GA LTHA and GA LTCA were absent during the mycelium growth process. Furthermore, the inactivated *G. sessile* failed to catalyze DHT to generate GAs. Additionally, enzymes extracted from the biotransformed mycelium demonstrated the ability to catalyze DHT and produce GA LTHA and GA LTCA.

Since the substrate DHT is a diterterpene structure, GA LTHA and GA LTCA may not be biosynthesized by the MVA pathway, and the transcriptome and proteome of *G. sessile* showed that there was no significant difference in the expression of key enzymes of MVA pathway at mRNA and protein levels, which further confirmed that triterpenoid GAs could be biotransformation from exogenous diterpenoid DHT. The GO analysis results showed that both DEGs and DEPs were enriched in FDH activity and oxidoreductase activity, suggesting that the biotransformation from DHT to GA may be related to the ROS in the mycelium increase. ROS was reported to play an important role in the growth of *G. lucidum* mycelium and GAs biosynthesis [23]. For examples, water stress [24], heat stress [25] and copper stress [26] can increase the ROS level in *G. lucidum* and positively regulate the biosynthesis of GA. In the differential protein KEGG pathway, the most significant cellular process enriched is the peroxisome, which is a special type of diverse microbodies, mainly containing oxidases, CAT and peroxidases to metabolize H_2_O_2_, thus regulating the ROS level [27]. Therefore, it is deduced that adding DHT may promote the ROS level in *G. sessile*, which boosts the mycelium to produce GAs due to the changing growth environment.

In addition, some proteins such as CYP450 (Gr-DN3779), FDH (Gr-DN9297, Gr-DN640) and CAT (Gr-DN3060) were upregulated at transcriptional and translational levels. As a monooxygenase, various chemical reactions can be catalyzed by CYP450 (eg, hydroxylation, demethylation, epoxidation), and some CYPs can modify different sites of one substrate [28]. For example, during the conversion of lanosterol to GA HLDOA, CYP5150L8 oxidizes the methyl group at C-26 of lanosterol to a hydroxyl, then the hydroxyl is oxidized to formyl and finally to carboxyl [10]. Besides, transferring electrons from NAD(P)H is usually required by CYP450 to facilitate the catalyzation of reaction [29]. If the transfer of electron is insufficient, more byproducts will be produced in the CYP catalyzed reaction, resulting in leakage of reducing equivalents and ultimately affect the activity of CYP [30].

**Fig. 8:**
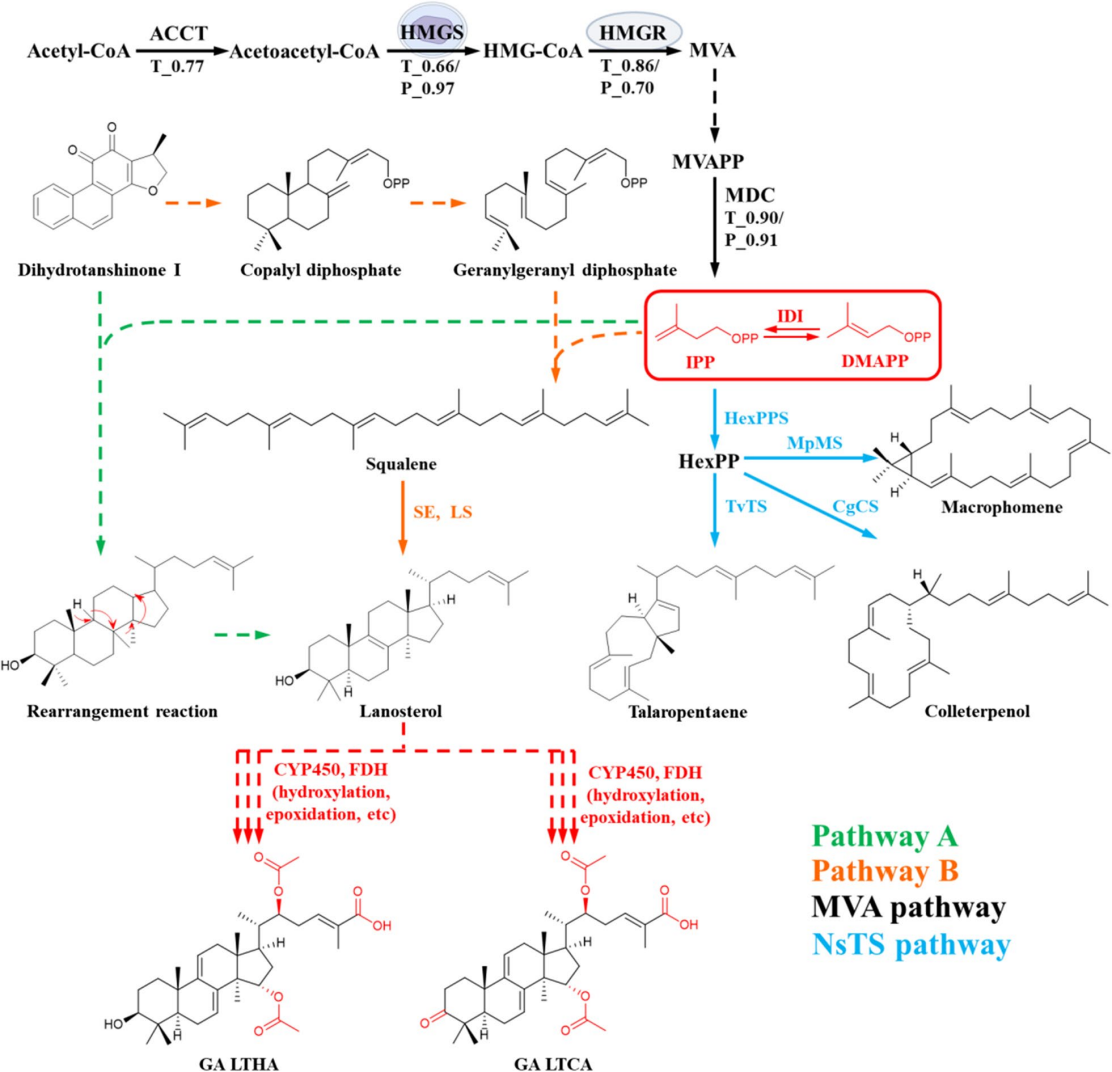
Proposed biosynthetic pathway of GAs from DHT in *G. sessile* mycelium. T and P represent fold changes in transcriptome and proteome, respectively; HexPPS, hexaprenyl diphosphate synthase; TvTS, talaromyces verruculosus talaropentaene synthase; MpMS, macrophomina phaseolina macrophomene synthase; CgCS, colletotrichum gloeosporioides colleterpenol synthase

The transcriptional and protein levels of FDH (Gr-DN9297 and Gr-DN640) were up-regulated by 4.95/1.74 and 2.36/1.68 times, respectively, and the catalytic efficiency of CYP (Gr-DN3779) was guaranteed by providing electrons. In addition, the upregulated expression of coenzymes, transporters and unrecognized proteins at the RNA and protein levels may play an important role in biotransformation of DHT to GAs. In Fig. 8, a biotransformation mechanism of GAs from DHT was proposed [31, 32]. Gene or protein expressions of enzymes that are implicated in the biosynthesis of GAs from lanosterol, especially CYP450, were significantly upregulated. It can be confirmed that lanosterol is the precursor in the biotransformation of GAs and DHT. In one possible route (Pathway A), more carbon atoms can be induced to the skeletal structure of DHT under the effect of IPP and DMAPP and lanosterol can be formed through rearrangement reaction. In another possible route (Pathway B), ring-opening reaction should be involved so that DHT can be first transformed into its biosynthetic precursor copalyl diphosphate and geranylgeranyl diphosphate. And the carbon chain is elongated by the IPP and DMAPP to form squalene and yield the lanosterol sequentially. Recently, a new enzymatic mechanism for triterpene biosynthesis has been reported through a non-squalene-dependent triterpene biosynthesis pathway (NsTS pathway) [33]. In our case, the biotransformation is less likely to occur through the latest reported pathway. At present, the relationship of structural changes between diterpene structures and triterpene structures has been rarely studied, so the underlying mechanisms need to be further elucidated.

In the market, the commercial cost of natural GAs is high because the GAs can generally only be found in the fruiting bodies and spores of *Ganoderma* [34–36]. It is of great potential to apply the biotransformation technology for industrial production [37]. Liquid fermentation is suitable for industrial production, and the yield of GAs can also be optimized by controlling fermentation conditions and exogenous elicitors. As compared to the biotransformation technology in laboratory, liquid fermentation may provide convenient to the application of GAs by shortening the incubation time for biotransformation and reducing the production cost.

## Conclusion

To conclude, two rare GAs, GA LTHA and GA LTCA, were obtained through biotransformation by adding the diterpenoid substrate DHT to the liquid culture of *G. sessile* mycelium. During 30 days of culture on a constant temperature shaker, the amount of DHT decreased while the content of GA LTHA and GA LTCA gradually increased. Transcriptomic and proteomic were employed to explore the biotransformation mechanism of GAs from exogenous diterpene DHT in the cultures of *G. sessile* mycelium. The data revealed that the number of DEGs and DEPs identified were 492 (213 upregulated and 279 downregulated) and 94 (52 upregulated and 42 downregulated), respectively. Results showed that transcription were positively correlated with the levels of protein expression. The addition of DHT expressively upregulated the bioactivities of formate dehydrogenase (NAD^+^) and oxidoreductase. And the expression enzymes including CYP450, FDH and CAT were also increased significantly, which may be involved in the structural transformation of DHT to GA LTHA and GA LTCA. This study confirmed that *G. sessile* mycelium in liquid culture can convert the diterpene DHT to the triterpene GAs. However, the underlying mechanism of biotransformation from diterpenes to triterpenes is seldomly reported, which is worth further investigation. The structure and biosynthetic pathway of triterpenes are usually complicated, and the extraction cost is high. Our findings provide new insights into the biotransformation of natural products by fungus, which will contribute to develop more efficient and economical ways to obtain the rare triterpenes in a convenient way.

## Materials and method

### Materials

*Ganoderma sessile* was obtained from Zhuhai Campus of Zunyi Medical University. Mold liquid medium and modified Martin agar were purchased from Guangdong Huankai Microbial Sci. & Tech. Co., Ltd (Guangdong, China). DHT was purchased from Chengdu Alfa Biotechnology Co., Ltd (Chengdu, China). Ethyl acetate, Petroleum ether, Sodium lauryl sulfate (SDS), Dithiothreitol (DTT), Urea, Trifluoroacetic acid (TFA), Indole-3-acetic acid (IAA), Tris–HCl, Methanol, Formic acid, Acetonitrile and Phosphate buffered saline (PBS) were purchased from Merck (Darmstadt, Germany).

### Methods of experimentation

#### Biotransformation and structural characterization

*G. sessile* was maintained in modified Martin agar at 4 °C before use. For activation, appropriate amount of mycelium was transferred to 200 mL of mold liquid medium, followed by breaking with a DREMEL Tissue-Tearor (Stuttgart, Germany) at 15,000 rpm. Mycelium was then cultured at 28 °C for 7 days at 200 rpm with shaking. For biotransformation, DHT was dissolved in anhydrous ethanol (1 mg/mL), then the solution was added to mycelium culture to achieve a DHT concentration of 40 mg/L. For control group, the medium was mixed with 8 mL of anhydrous ethanol without DHT. Then, the mycelium was then cultured in a shaker for 30 days at 200 rpm and 28 °C. For every 5 days, 10 mL culture medium was collected for centrifugation and separation. Mycelium was added to methanol for ultrasonic treatment. Mycelium and upper solution were evaporated and dried, respectively, and methanol was redissolved. After that, the composition and content changes in mycelium and supernatant were detected by UPLC (Waters, MA, USA) using a BEH Shield RP18 (2.1 × 100 mm, 1.7 μm, Waters) with an injection volume of 2 μL and detection wavelengths at 260 nm with column temperature at 40 °C. Gradients of solvent A (acetonitrile) and solvent B (0.1% formic acid in water) were prepared as follows (V/V): (I) 0–2 min (A: B, 3/7), (II) 2–7 min (A: B, 5/5), (III) 7–10 min (A: B, 7/3), (IV) 10–11 min (A: B, 10/0), (V) 11–12 min (A: B, 3/7) with a flow rate of 0.3 mL/min. In order to further verify the specificity of DHT biotransformation by *G. sessile*, the mycelium and enzymes in the culture medium were inactivated by boiling water bath at 100 °C for 20 min. Substrate DHT (40 mg/L) was added into the deactivated medium, cultured in a shaker according to the experimental method described above. And the components in the samples were detected by UPLC.

The intracellular and extracellular enzyme activities of *G. sessile* before and after biotransformation were compared. Extracellular enzyme was the supernatant collected by vacuum filtration from the culture medium. According to previous reports, intracellular enzymes were extracted from mycelium [38]. In short, the same volume of PBS (20 mL) was added to the mycelium collected from the culture medium. Mycelium was breaking with a DREMEL Tissue-Tearor for 1 min and rested for 1 min, repeated three times, after which it was treated with an ultrasound device for 3 min. The above steps were performed in an ice water bath. The crude extract was centrifuged at 5000 rpm for 10 min at 4 °C, and the upper layer was taken as the extracellular enzyme. Then 45 μL DHT solution (1 mg/mL) was added to 1.5 mL extract of intracellular and extracellular enzymes, respectively. The mixture was incubated at 250 rpm and 28 °C for 72 h, and the changes in composition were detected by UPLC.

#### Isolation and identification of biotransformation products

To separate the biotransformation products, contents in mycelium were extracted using 80% methanol, dichloromethane and water with ultrasonic. The solvent was evaporated at vacuum to yield the crude extract, and then dissolved in methanol. Each component in the crude extract was separated by silica gel column chromatography using ethyl acetate/petroleum ether as eluent. Next, the structures of extracted compounds were characterized by using high-resolution electrospray ionization mass spectrometry (HR-ESI–MS, Q-Exactive, Thermo Fisher, MA, USA), NMR spectrometer (Bruker Avance III, Bruker Corporation, Germany).

#### RNA isolation, library construction, and sequencing

Samples of *G. sessile* mycelium is ground into a powder in liquid nitrogen, and the total RNA was extracted from mycelium using MPFast RNA Red Kit (MP Biomedicals, CA, USA). cDNA library construction and transcriptome sequencing were conducted by Bioprofile Technology Co., Ltd. (Shanghai, China, http://www.bioprofile.cn).

#### Protein digestion and TMT labeling of peptides

Protein extraction, digestion and peptide TMT labeling of *G. sessile* according to previous report [39], and then the TMT labeled sample are analysis by LC–MS/MS.

#### LC–MS/MS detection and database search analysis

Samples were purified using an Easy-nLC 1200 system (Thermo Fisher, MA, USA) with a trap column (100 µm × 20 mm, 5 µm, C18, Thermo Fisher), and then passed through an EASY analysis column (75 µm × 150 mm, 3 µm, C18, Thermo Fisher) for separation. 0.1% formic acid in water and 0.1% formic acid in acetonitrile were used as mobile phase. The flow rate was 300 nL/min with column temperature of 40℃. Mass spectrometry analysis was performed with a Q-Exactive HF-X mass spectrometer (Thermo Fisher) with precursor ion scanning range from 350 to 1800 m/z. The resulting LC–MS/MS raw files were imported into Proteome Discoverer 2.4 software (Thermo Fisher) for analysis and the database used for protein identification was derived from the transcriptome sequencing results of *G. sessile* mycelium, the Additional file 1: Table S3 shows the main search parameters. TMT reporter ion intensity was used for quantification.

#### Bioinformatics analysis

Analyses of bioinformatics data were carried out with Perseus software [40], Microsoft Excel and R statistical computing software. Comparative transcriptome analysis and proteome analysis were carried out with the cutoff of a ratio |log_2_ FC| of > 1 or |FC| of > 1.2 with *p* < 0.05, respectively. Information was extracted from UniProtKB/Swiss-Prot [41], Kyoto Encyclopedia of Genes and Genomes (KEGG) [42], and Gene Ontology (GO) to annotate sequences. In addition, subcellular localization of DEPs is annotated and counted by GO database [43, 44].

#### Gene expression analysis by RT-qPCR

RNA was extracted from *G. sessile* mycelium of experimental (E) and control (C) groups and used for RT-qPCR. Fungal 18S rRNA was used as an internal reference gene [45, 46], and Gene-specific primers for RT-qPCR are shown in Additional file 1: Table S4. Three technical repeats were performed. The reaction consisted of the following two steps: RT-PCR (reverse transcription PCR) was performed using the All-in-one RT SuperMix Perfect for qPCR (Vazyme, Jiangsu, China), and the qPCR was performed using the ChamQ Universal SYBR qPCR Master Mix (Vazyme), RT-PCR and qPCR procedures followed the kit steps respectively. The RT-qPCR data were analyzed by the comparative delta-delta Ct method (2^−ΔΔCt^) for a relative quantification of the amplicons [47].

## Supplementary Information

**Additional file 1**: **Figure S1.** UPLC chromatograms of C group *G. sessile* biotransformation before and after 30 days; **Figure S2.** Separation and purification of compounds and structural characterization; **Figure S3.** Separation and purification of compounds and structural characterization; **Table S1.** ^1^H (600 MHz) and ^13^C (150 MHz) NMR spectral data of compounds 1 and 2 (δ in ppm, *J* in Hz); **Table S2.** Transcriptome sequencing results of *G. sessile*; **Table S3.** Proteome Discoverer database search parameters; **Table S4.** Gene-specific primers used for RT-qPCR.

## Data Availability

The raw sequence data have been deposited in the NCBI Sequence Read Archive (SRA) database (BioProject: PRJNA940961 and Submission: SUB12932487). The mass spectrometry proteomics data have been deposited to the PRIDE partner repository with the data-set identifier PXD040674.

## Abbreviations

BP: Biological process
CAT: Catalase
CC: Cellular component
CYP450: Cytochrome P450 enzyme
DEG: Differentially expressed gene
DEP: Differentially expressed protein
DHT: 15,16-Dihydrotanshinone I
DMAPP: Dimethylallyl diphosphate
FDH: NAD-dependent formate dehydrogenase
GA: Ganoderic acid
MF: Molecular function
IPP: Isopentenyl diphosphate
LTCA: Lanosta-7,9(11),24-trien-15*α*,22,*β*-diacetoxy-3*β*-carbonyl-26-oic acid
LTHA: Lanosta-7,9(11),24-trien-15*α*,22,*β*-diacetoxy-3*β*-hydroxy-26-oic acid
MEP: 2-C-methyl-d-erythritol 4-phosphate
MF: Molecular function
MVA: Mevalonate
NCBI: National Center for Biotechnology Information
UPLC: Ultra performance liquid chromatography
