# Supplementary material for: Biotransformation of triterpenoid ganoderic acids from exogenous diterpene dihydrotanshinone I in the cultures of Ganoderma sessile
Source: Microb Cell Fact. 2023 Jul 28;22:139. doi: 10.1186/s12934-023-02156-5 (PMC10375632; doi:10.1186/s12934-023-02156-5)
Supplement: Supplementary file 1 — Additional file 1: Figure S1. UPLC chromatograms of C group G. sessile biotransformation before and after 30 days; Figure S2. Separation and purification of compounds and structural characterization; Figure S3. Separation and purification of compounds and structural characterization; Table S1. 1H (600 MHz) and 13C (150 MHz) NMR spectral data of compounds 1 and 2 (δ in ppm, J in Hz); Table S2. Transcriptome sequencing results of G. sessile; Table S3. Proteome Discoverer database search parameters; Table S4. Gene-specific primers used for RT-qPCR. [file 12934_2023_2156_MOESM1_ESM.docx]

Additional file 1 to

**Biotransformation of Triterpenoid Ganoderic Acids from Exogenous Diterpene Dihydrotanshinone I in the Cultures of *Ganoderma sessile***

Xinwei Wang^a^†, Haibo Wu ^a^†, Ka Hong Wong^b^†, Yixuan Wang^a^, Bai-Xiong Chen^a^, Kun Feng^a*^

*^a^ School of Bioengineering, Zhuhai Campus of Zunyi Medical University, Zhuhai, Guangdong, China.*

*^b^ State Key Laboratory of Quality Research in Chinese Medicine, Institute of Chinese Medical Sciences, University of Macau, Macau, China.*

*Corresponding author: Kun FENG, [fengk@zmu.edu.cn](mailto:fengk@zmu.edu.cn)

**Figure S1:** **UPLC chromatograms of C group *G. sessile* biotransformation before and after 30 days.**

**
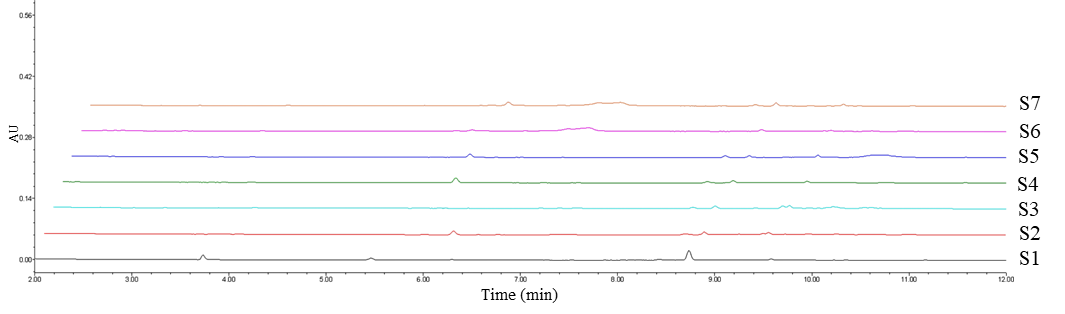
**

**Fig. S1.1** UPLC chromatogram of mycelium extract of C group^[[1]](#footnote-1)^


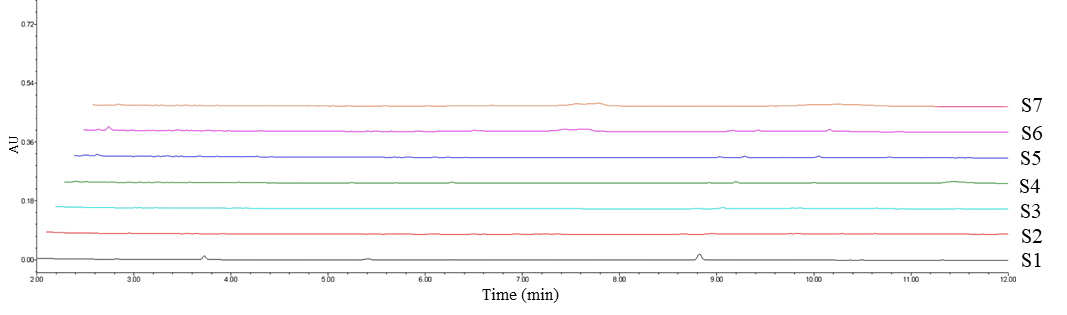


**Fig. S1.2** UPLC chromatogram of culture medium extract of C group

**Figure S2: Biotransformation of DHT by enzyme extracted from *G. sessile*.**

**
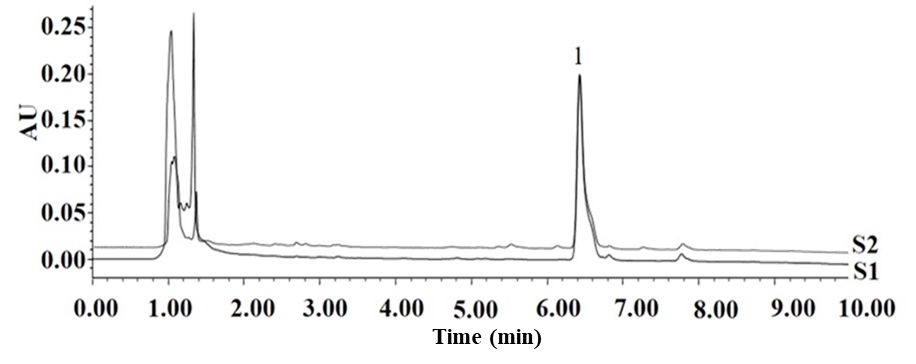
**

**Fig. S2.1** Intracellular enzymes of *G. sessile^[[2]](#footnote-2)^*

**
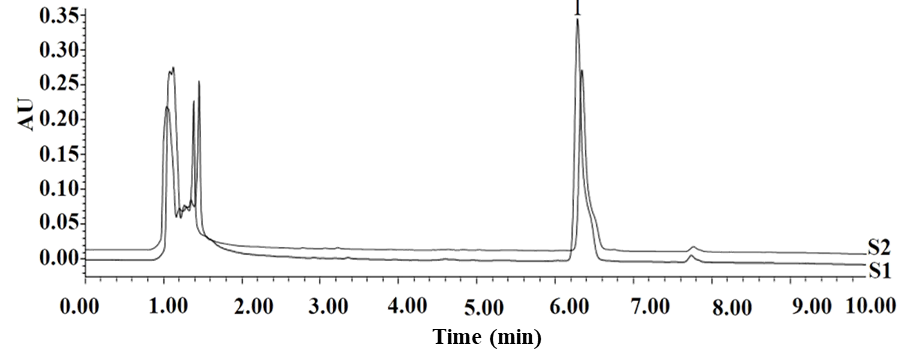
**

**Fig. S2.2** Extracellular enzymes of *G. sessile*

**
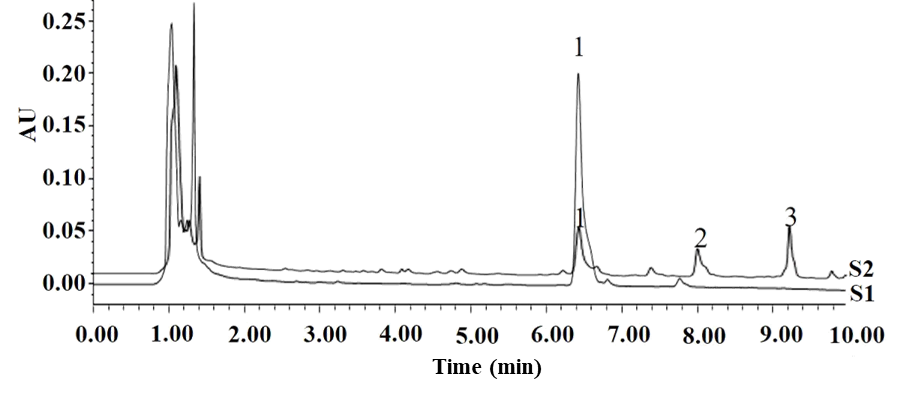
**

**Fig. S2.3** Intracellular enzymes of *G. sessile* after biotransformation

**
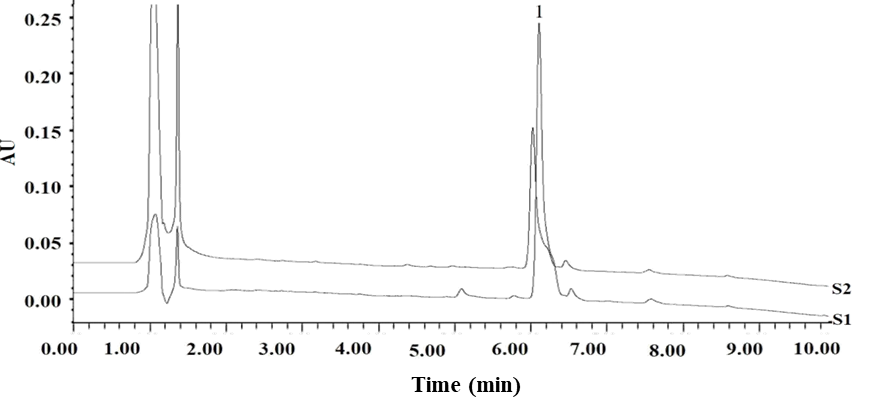
**

**Fig. S2.4** Extracellular enzymes of *G. sessile* after biotransformation

**Figure S3: Separation and purification of compounds and structural characterization.**


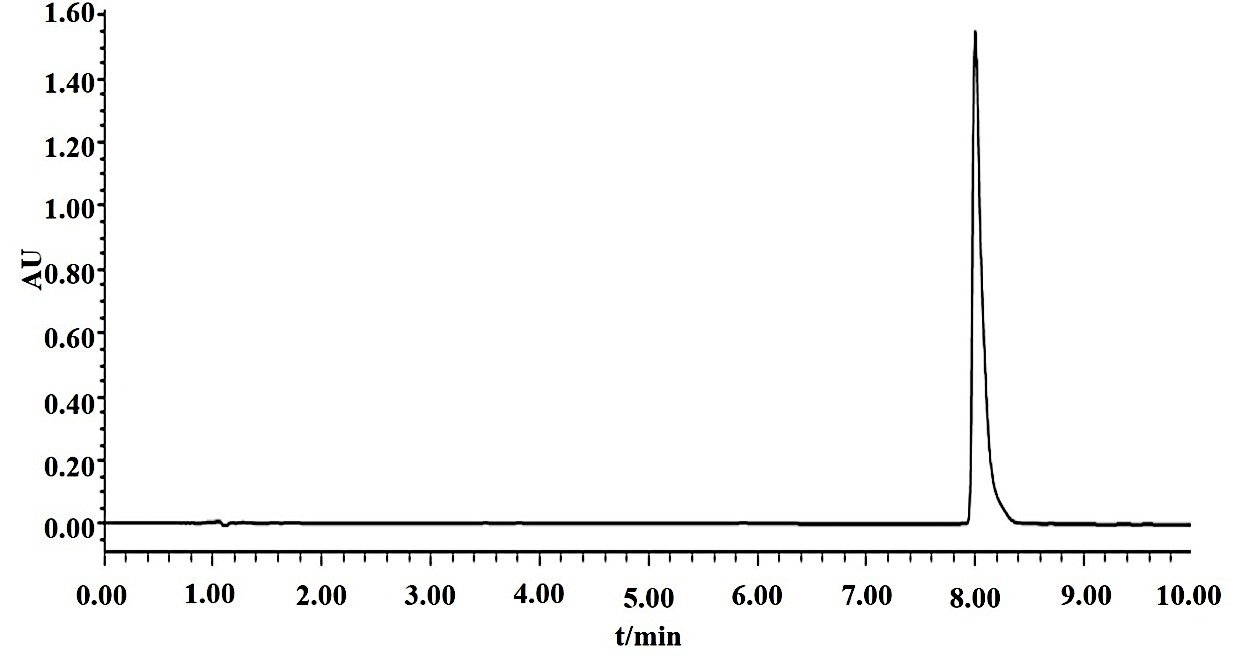


**Fig. S3.1** The UPLC chromatogram profile of compound 1


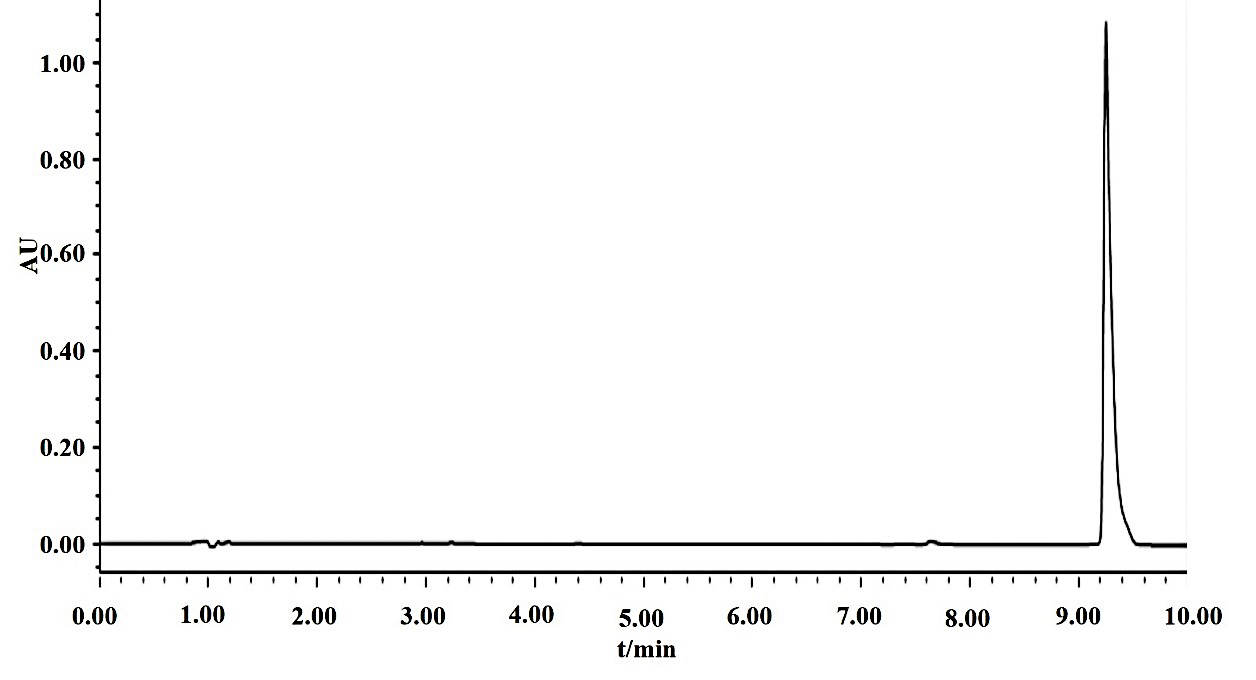


**Fig. S3.2** The UPLC chromatogram profile of compound 2


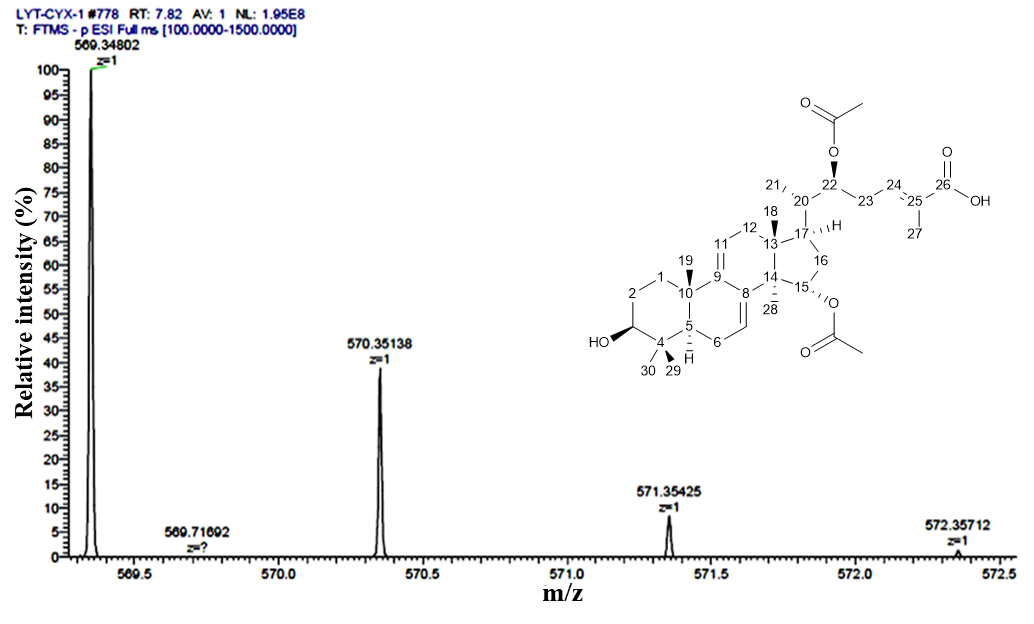


**Fig. S3.3** High-resolution mass spectrum of compound 1


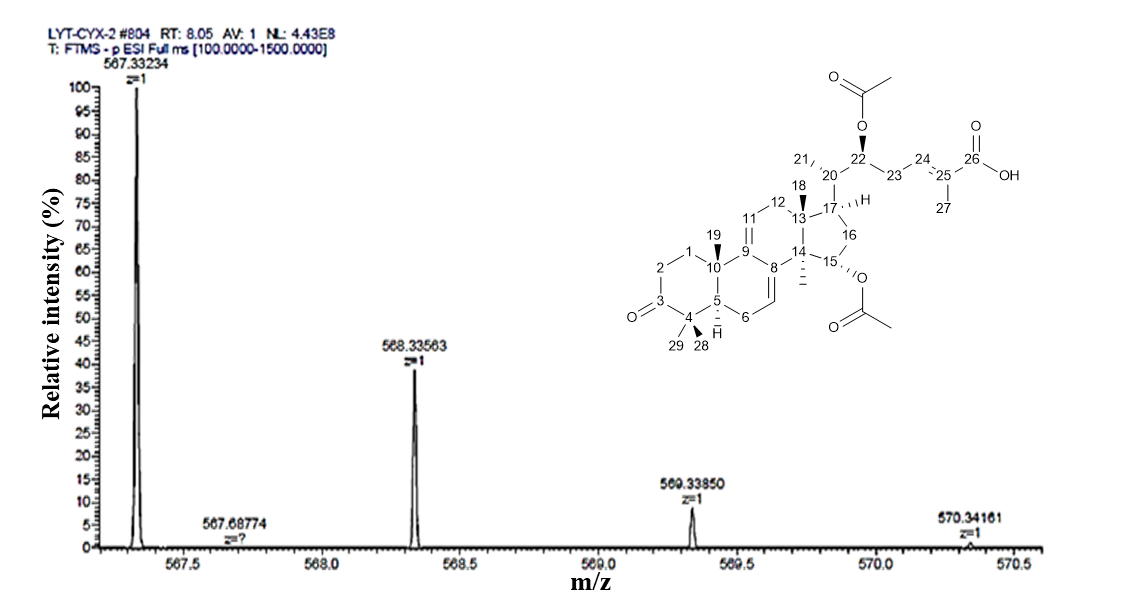


**Fig. S3.4** High-resolution mass spectrum of compound 2


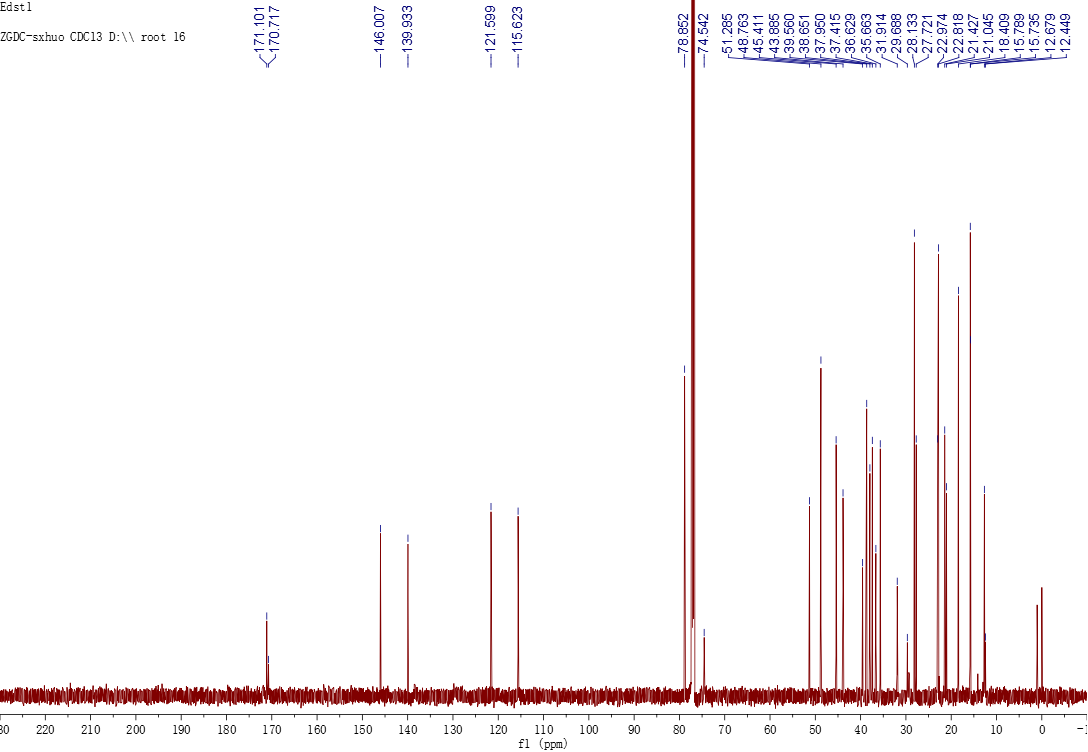


**Fig. S3.5** ^13^C-NMR spectrum of compound 1 (CDCl_3_, 150 MHz)


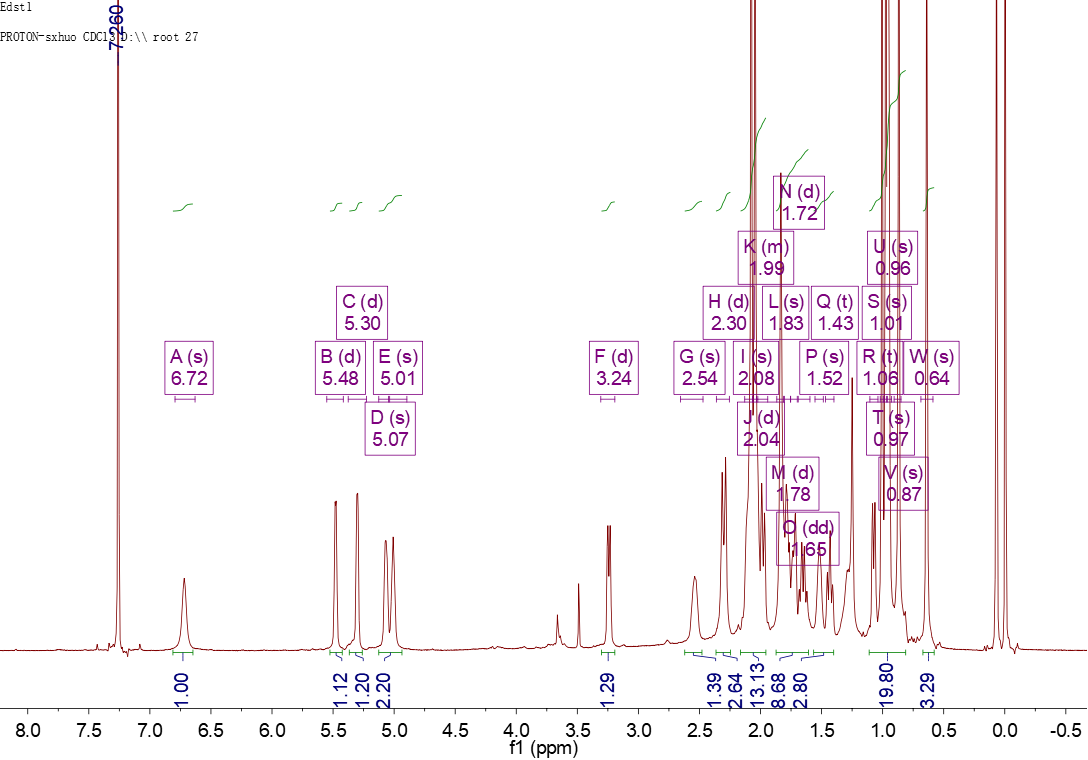


**Fig. S3.6** ^1^H-NMR spectrum of compound 1 (CDCl_3_, 600 MHz)


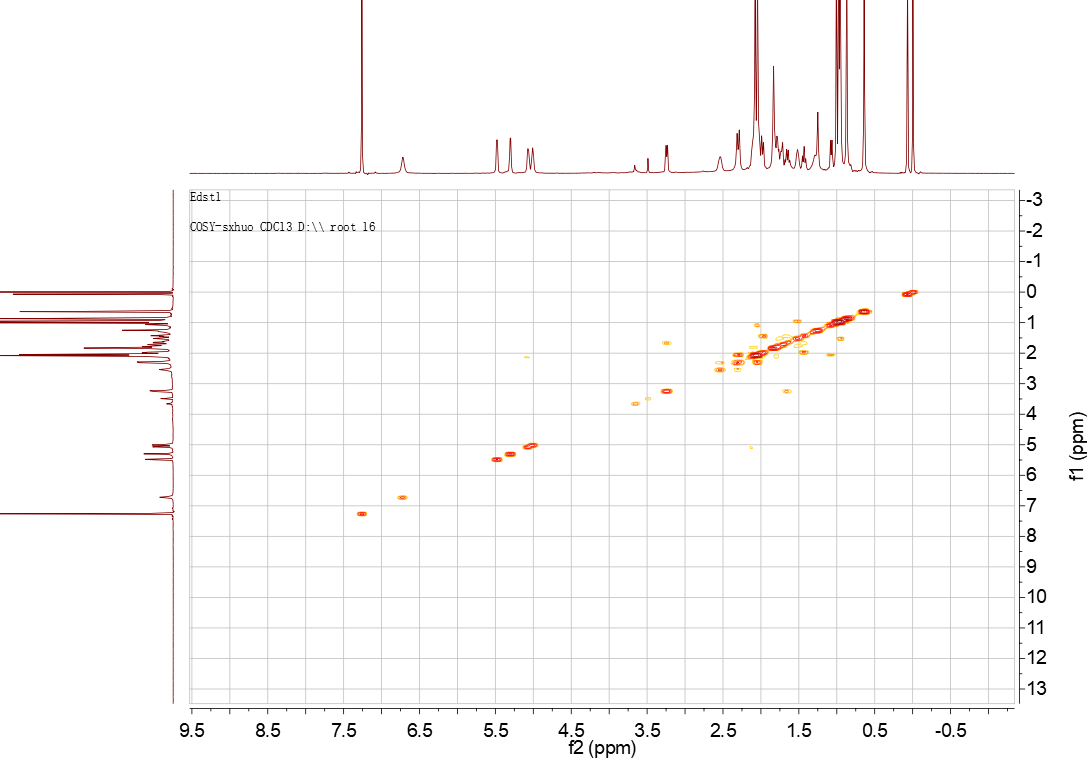


**Fig. S3.7** ^1^H-^1^HCOSY spectrum of compound 1


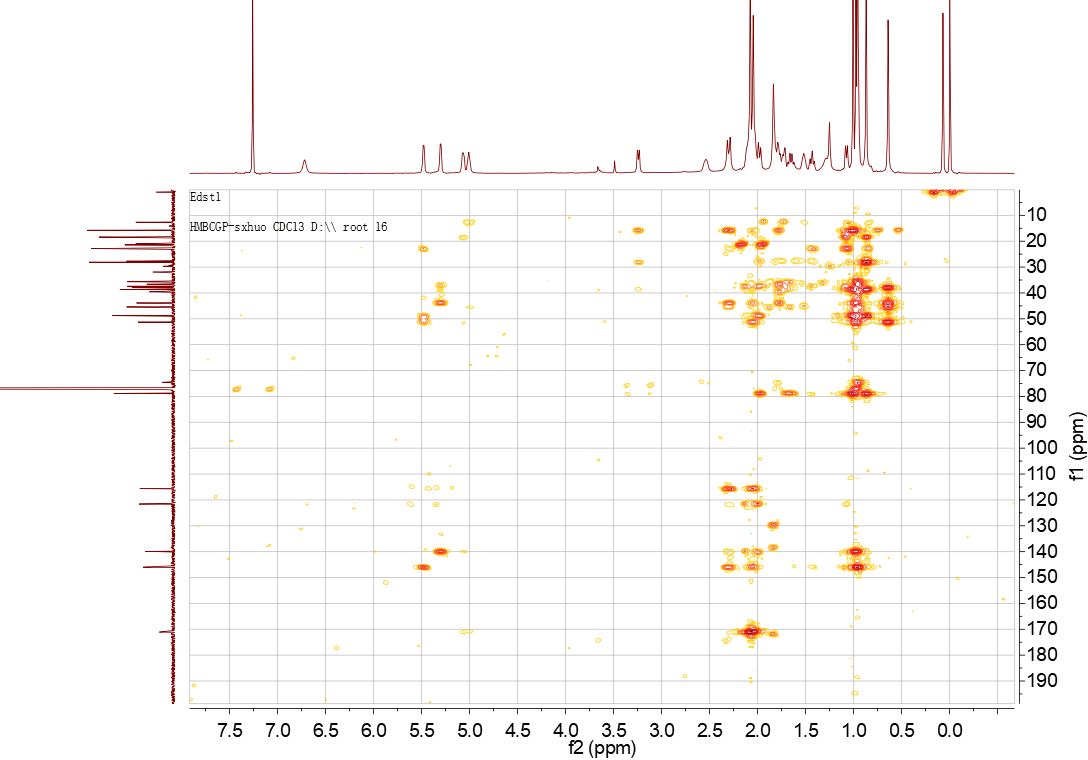


**Fig. S3.8** HMBC spectrum of compound 1


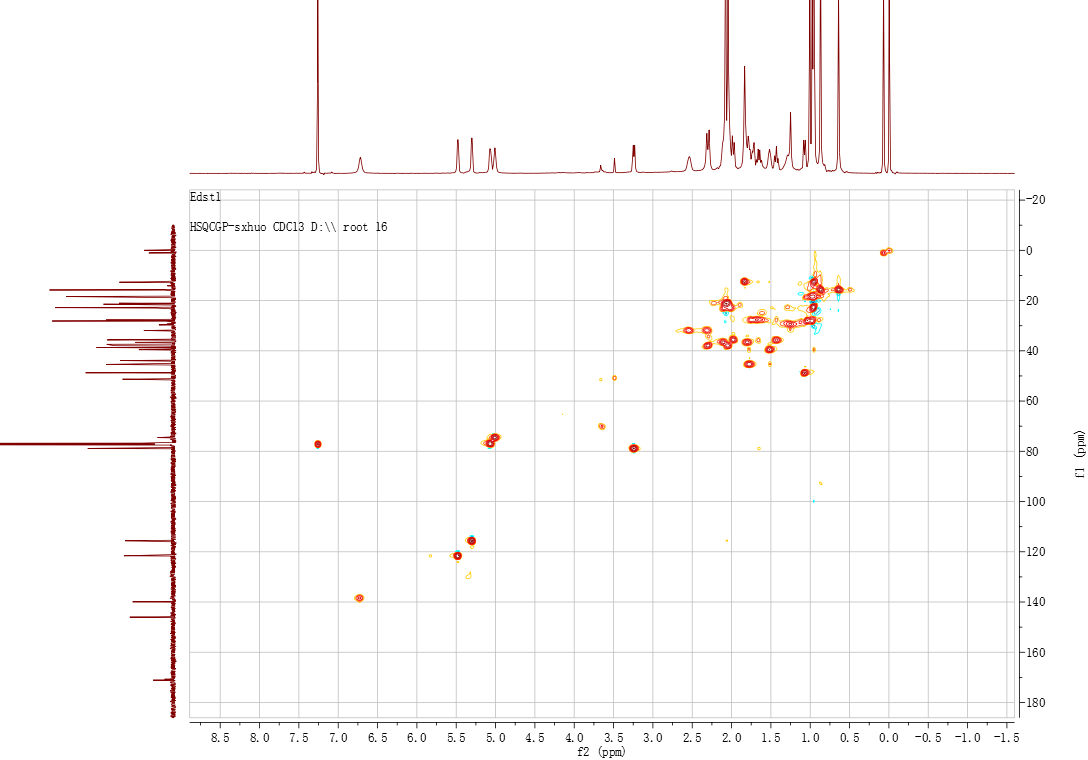


**Fig. S3.9** HSQC spectrum of compound 1


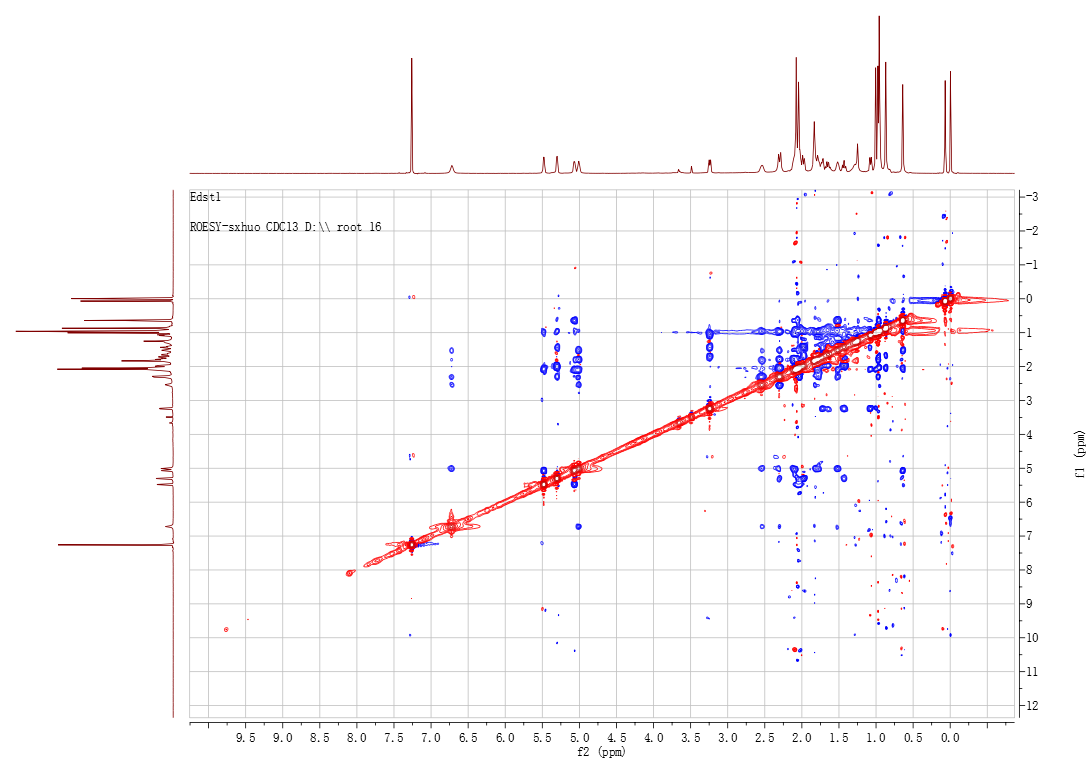


**Fig. S3.10** ROESY spectrum of compound 1


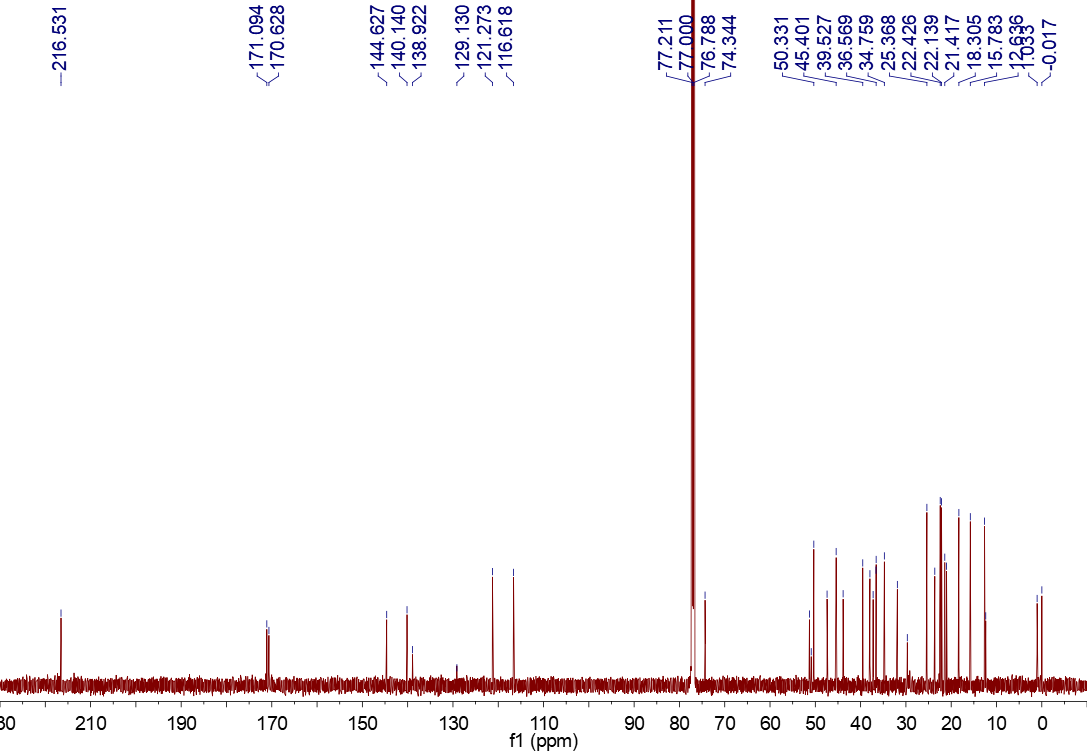


**Fig. S3.11** ^13^C-NMR spectrum of compound 2 (CDCl_3_, 150 MHz)


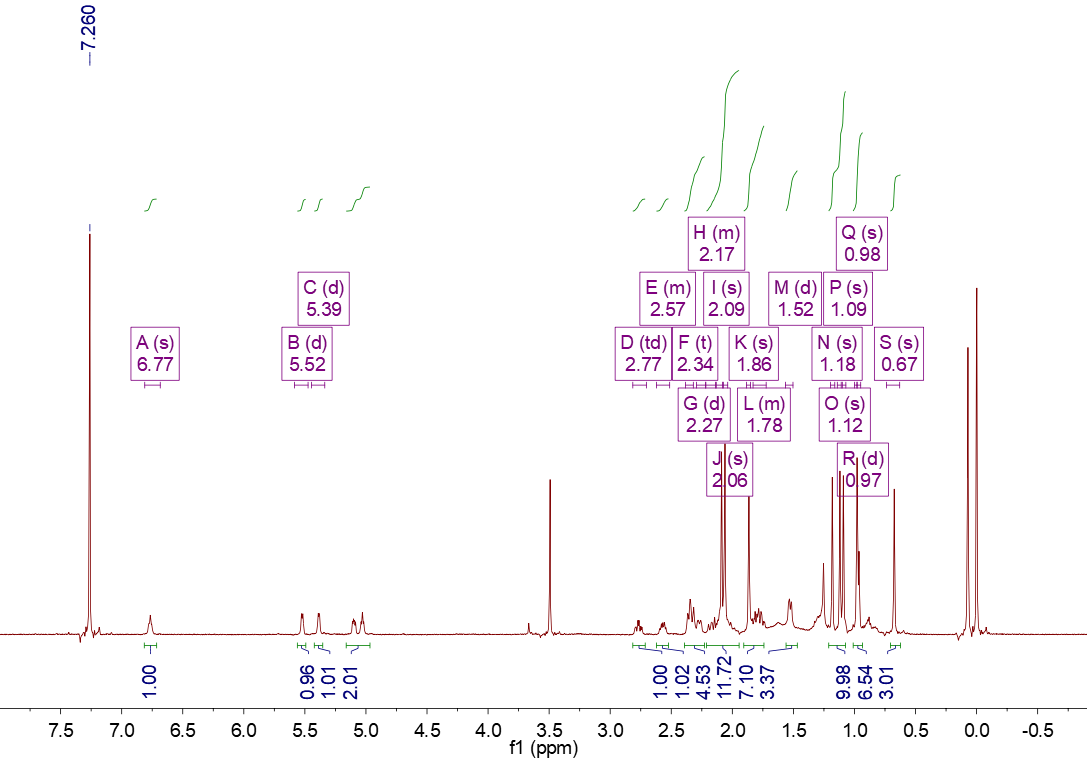


**Fig. S3.12** ^1^H-NMR spectrum of compound 2 (CDCl_3_, 600 MHz)


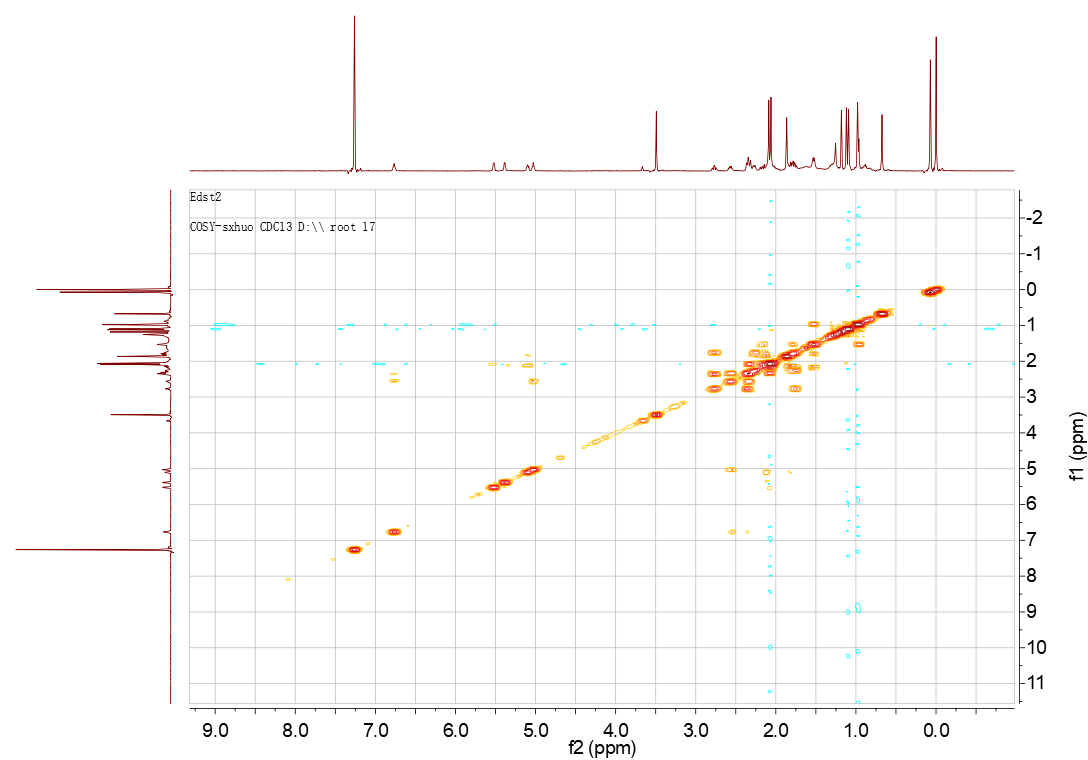


**Fig. S3.13** ^1^H-^1^HCOSY spectrum of compound 2

**
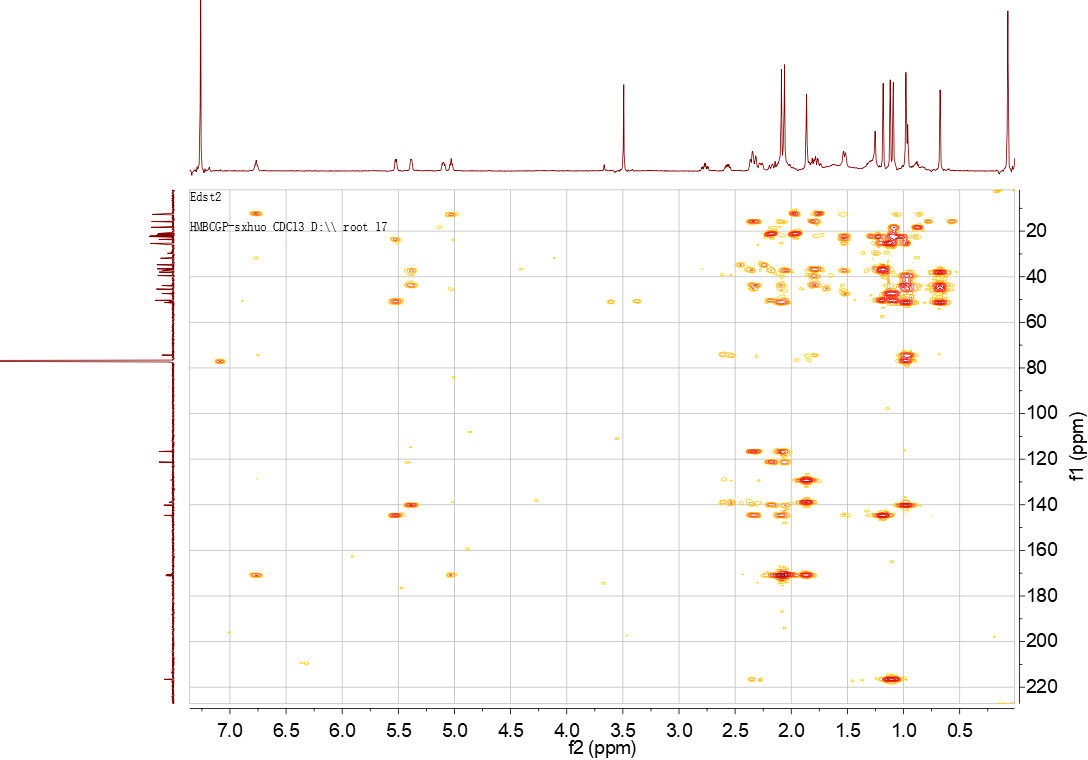
**

**Fig. S3.14** HMBC spectrum of compound 2


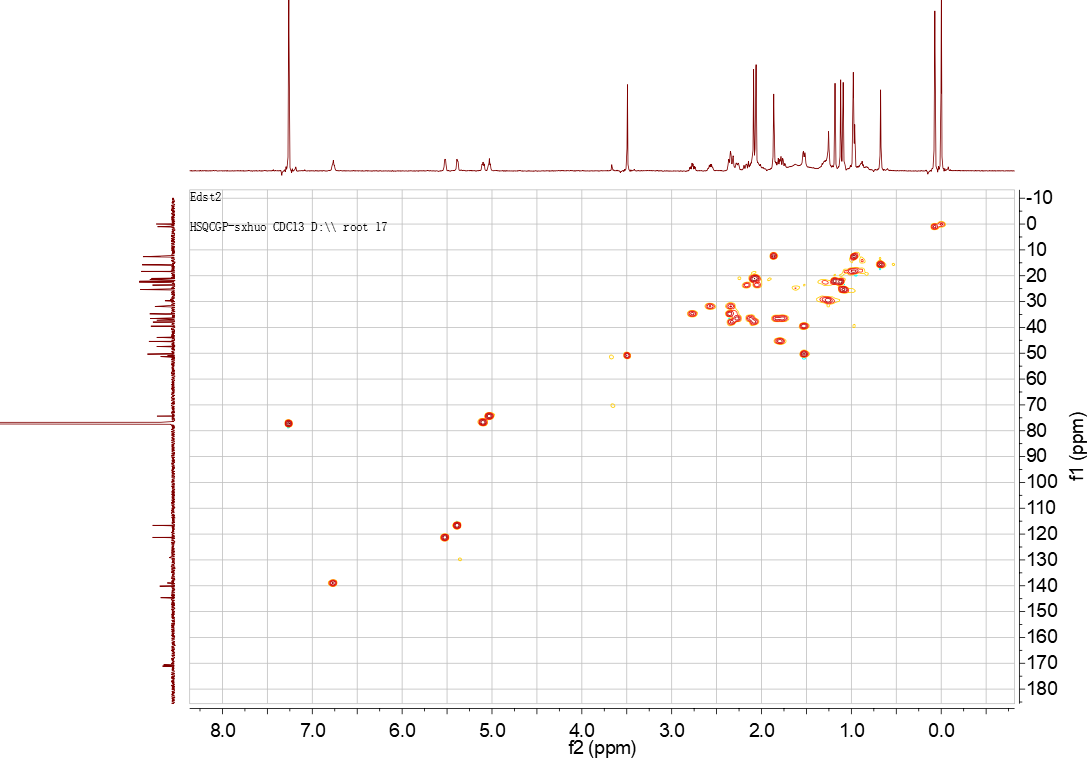


**Fig. S3.15** HSQC spectrum of compound 2


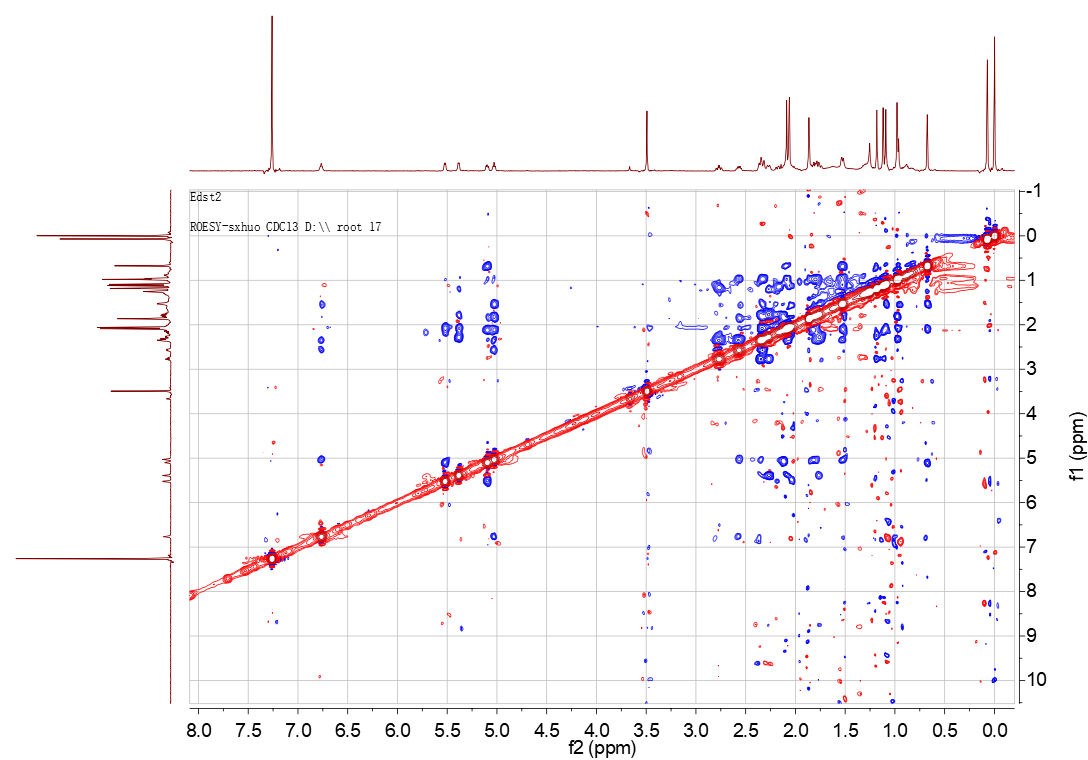


**Fig. S3.16** ROESY spectrum of compound 2

**Table S1:** **^1^H (600 MHz) and ^13^C (150 MHz) NMR spectral data of compounds 1 and 2 (δ in ppm, *J* in Hz).**

| **No.** | **Compounds 1** | | **Compounds 2** | |
| --- | --- | --- | --- | --- |
|  | **δ_C_** | **δ_H_** | **δ_C_** | **δ_H_** |
| 1 | 35.7 | 1.98 (brd J=13.8) | 36.6 | 2.28 (d) 1.76 (m) |
|  |  | 1.43 (brt J=13.6) |  |  |
| 2 | 27.7 | 1.67 (dd)1.00 (s) | 34.8 | 2.77 (td) 2.35 (m) |
| 3 | 78.9 | 3.24 (brd J=11.4) | 216.5 |  |
| 4 | 38.7 |  | 47.4 |  |
| 5 | 48.8 |  | 50.3 |  |
| 6 | 23.0 | 2.11-2 | 23.6 | 2.16 (m) 2.04 (s) |
| 7 | 121.6 | 5.48 (d J=4.5) | 121.3 | 5.52 (d) |
| 8 | 139.9 |  | 140.1 |  |
| 9 | 146.0 |  | 144.6 |  |
| 10 | 37.4 |  | 37.2 |  |
| 11 | 115.6 | 5.30 (d J=4.5) | 116.6 | 5.39 (d) |
| 12 | 38.0 | 2.30 (d) 2.05 (d) | 37.9 | 2.33 (t )2.08 (s) |
| 13 | 43.9 |  | 43.9 |  |
| 14 | 51.3 |  | 51.3 |  |
| 15 | 76.9 | 5.07 (s w1/2=18.4) | 76.8 | 5.10 (dd J=9.5 5.0) |
| 16 | 36.6 | 2.10 (s) 1.80 (d) | 36.6 | 2.10 (s) 1.80 (m) |
| 17 | 45.4 | 1.76 (d) | 45.4 | 1.79 (m) |
| 18 | 15.8 | 0.64 (s) | 15.8 | 0.68 (s) |
| 19 | 22.8 | 0.96 (s) | 22.1 | 1.18 (s) |
| 20 | 39.6 | 1.51 (s) | 39.5 | 1.53 (d) |
| 21 | 12.7 | 0.95 (s) | 12.6 | 0.97 (d) |
| 22 | 74.5 | 5.01 (s w1/2=15.7) | 74.3 | 5.03 (t) |
| 23 | 31.9 | 2.54 (s) 2.30 (d) | 31.9 | 2.57 (m) 2.35 (t) |
| 24 | 138.4 | 6.72 (s w1/2=21.7) | 138.9 | 6.77 (t J=7.0) |
| 25 | 129.6 |  | 129.1 |  |
| 26 | 171.8 |  | 170.8 |  |
| 27 | 12. 4 | 1.83 (s) | 12.4 | 1.86 (s) |
| 28 | 18.4 | 0.98 (s) | 25.4 | 1.09 (s) |
| 29 | 28.1 | 1.01 (s) | 22.4 | 1.12 (s) |
| 30 | 15.7 | 0.87 (s) | 18.47 |  |
| 15-OCOCH_3_ | 171.1 |  | 171.1 |  |
| 15-OCOCH_3_ | 21.4 | 2.06 (3H s) | 21.4 | 2.09 (3H s) |
| 22-OCOCH_3_ | 170.7 |  | 170.6 |  |
| 22-OCOCH_3_ | 21.0 | 2.05 (3H s) | 21.0 | 2.06 s (3H s) |

**Table S2:** **Transcriptome sequencing results of *G. sessile*.**

|  | **Transcript** | **Unigene** |
| --- | --- | --- |
| Total length (bp) | 146876180 | 28098155 |
| Sequence number | 67230 | 14298 |
| Max. length (bp) | 15424 | 15424 |
| Mean. length (bp) | 2184.68 | 1965.18 |
| N50 (bp) | 2910 | 2841 |
| N90 (bp) | 1198 | 1038 |
| GC% | 57.27 | 57.18 |

**Table S3: Proteome Discoverer database search parameters.**

| **Item** | **Value** |
| --- | --- |
| Type | Reporter ion MS2 |
| Isobaric labels | TMT6plex |
| Enzyme | Trypsin |
| Reporter mass tolerance | 0.005 Da |
| Max missed cleavages | 2 |
| Main search peptide tolerance | 10 ppm |
| MS/MS tolerance | 0.02 Da |
| Fixed modifications | Carbamidomethyl (C), TMT6plex (K), TMT6plex (Peptide N-term) |
| Variable modifications | Oxidation (M), Acetyl (Protein N-term) |
| Database | Unigene.fa.transdecoder.pep.fasta |
| Database pattern | Target-Reverse |
| PSM FDR | ≤0.01 |
| Protein FDR | ≤0.01 |
| Protein quantification | Razor and unique peptides were used for protein quantification |

**Table S4.** **Gene-specific primers used for RT-qPCR.**

| **Gene name** | **Forward primer (5’-3’)** | **Reverse primer (5’- 3’)** |
| --- | --- | --- |
| 18S rRNA | TATCGAGTTCTGACTGGGTTGT | ATCCGTTGCTGAAAGTTGTAT |
| PSD | CGAGTACGTTCACTGGGTCC | CCGACGGATCAATAGGGCTC |
| FDH | CCTGGGACACGAAGTCTTTCA | CTGCCGTTTAACCCCAAGGA |
| HPs2 | TACGTCAGTCACACACCAGC | GCGGTCGCAGAATATCCTCA |
| Catalase | ACTTTGTGGGTAACGTGGCT | GACTGGTCTACGGCAGCAAA |
| CYP450 | GAGAGATACTGAGCGTCGGC | TCACCTCTAAAGCCCCCACT |
| GLB | GGAGATGCGGTAGTGGTCTG | CACCCCGATCTCTGCTTACC |
| AST | GTCAGGTAGATGTGCGCCTT | ACACGGGCTTTGTTTGTTCG |
| Lipase | GCTGCACCTTCGTATGTCCT | TTGCGTCACCCCTCATGTTT |
| FDH2 | TACGACTACAACGCCCTTCC | GGCTAACGGAGACGTACCTG |
| HPs3 | GAGGGGCATCTCGTAGTTCG | GCATGGGGATGAAGCAGACT |
| TP | GTGTGGCAAAAGCAGACTCG | GGACAGCTAGTACGAACGGG |

1. S1 ~ S7: 1, 5, 10, 15, 20, 25 and 30 days of biotransformation, respectively. [↑](#footnote-ref-1)
2. S1, Enzyme catalyzed DHT for 1 h; S2, Enzyme catalyzed DHT for 72 h; 1, DHT; 2, GA LTHA; 3, GA LTCA. [↑](#footnote-ref-2)
